# Gout is associated with an increased risk for incident heart failure among older adults: the REasons for Geographic And Racial Differences in Stroke (REGARDS) cohort study

**DOI:** 10.1186/s13075-020-02175-2

**Published:** 2020-04-16

**Authors:** Lisandro D. Colantonio, Kenneth G. Saag, Jasvinder A. Singh, Ligong Chen, Richard J. Reynolds, Angelo Gaffo, Timothy B. Plante, Jeffrey R. Curtis, S. Louis Bridges, Emily B. Levitan, Ninad S. Chaudhary, George Howard, Monika M. Safford, Paul Muntner, Marguerite Ryan Irvin

**Affiliations:** 1 Department of Epidemiology, University of Alabama at Birmingham, 1720 2nd Ave South, RPHB 527C, Birmingham, AL 35294-0013 USA; 2 Division of Clinical Immunology and Rheumatology, University of Alabama at Birmingham, Birmingham, AL USA; 3 Birmingham Veterans Affairs Medical Center, Birmingham, AL USA; 4 Department of Medicine, Larner College of Medicine at the University of Vermont, Burlington, VT USA; 5 Department of Biostatistics, University of Alabama at Birmingham, Birmingham, AL USA; 6 Department of Medicine, Weill Cornell Medical College, New York, NY USA

**Keywords:** Gout, Heart failure, Cardiovascular disease, Risk factors

## Abstract

**Background:**

Gout has been associated with a higher risk for coronary heart disease (CHD) and stroke in some prior studies. Few studies have assessed the association of gout with incident heart failure (HF).

**Methods:**

We analyzed data from 5713 black and white men and women ≥ 65.5 years of age in the population-based REasons for Geographic And Racial Differences in Stroke (REGARDS) cohort study who had Medicare coverage without a history of HF, CHD, or stroke at baseline between 2003 and 2007. Gout was defined by ≥ 1 hospitalization or ≥ 2 outpatient visits with a diagnosis code for gout in Medicare claims prior to each participant’s baseline study examination. REGARDS study participants were followed for HF hospitalization, CHD, stroke, and all-cause mortality as separate outcomes through December 31, 2016. Analyses were replicated in a random sample of 839,059 patients ≥ 65.5 years of age with Medicare coverage between January 1, 2008, and June 30, 2015, who were followed through December 31, 2017.

**Results:**

Among REGARDS study participants included in the current analysis, the mean age at baseline was 72.6 years, 44.9% were men, 31.4% were black, and 3.3% had gout. Over a median follow-up of 10.0 years, incidence rates per 1000 person-years among participants with and without gout were 13.1 and 4.4 for HF hospitalization, 16.0 and 9.3 for CHD, 9.3 and 8.2 for stroke, and 55.0 and 37.1 for all-cause mortality, respectively. After multivariable adjustment for sociodemographic variables and cardiovascular risk factors, hazard ratios (95% CI) comparing participants with versus without gout were 1.97 (1.22, 3.19) for HF hospitalization, 1.21 (0.79, 1.84) for CHD, 0.83 (0.48, 1.43) for stroke, and 1.08 (0.86, 1.35) for all-cause mortality. The multivariable-adjusted hazard ratio for HF hospitalization with reduced and preserved left ventricular ejection fraction among participants with versus without gout was 1.77 (95% CI 0.83, 3.79) and 2.32 (95% CI 1.12, 4.79), respectively. The multivariable-adjusted hazard ratio for heart failure hospitalization associated with gout among the 839,059 Medicare beneficiaries was 1.32 (95% CI 1.25, 1.39).

**Conclusion:**

Among older adults, gout was associated with an increased risk for incident HF but not for incident CHD, incident stroke, or all-cause mortality.

## Background

Prior studies suggest that gout, an inflammatory disease caused by the deposit of monosodium urate crystals in joints and tissues [1], is associated with an increased risk for atherosclerotic cardiovascular disease, including coronary heart disease (CHD) and stroke [2–4]. Many risk factors for CHD and stroke, including hypertension, diabetes, cigarette smoking, and obesity, are also associated with an increased risk for heart failure (HF) [5]. If gout is associated with an increased risk for HF, this would support the need for interventions to prevent its occurrence in this population.

The objective of the current study was to determine the risk for incident HF associated with gout in a population-based cohort of black and white adults enrolled in the REasons for Geographic And Racial Differences in Stroke (REGARDS) study [6]. Additionally, we evaluated the association of gout with incident CHD, incident stroke, and all-cause mortality. We replicated the analysis in a random sample of Medicare beneficiaries to determine whether associations in the REGARDS study were also present in an independent study sample.

## Methods

The REGARDS study enrolled 30,239 black and white participants ≥ 45 years of age from all 48 contiguous US states and the District of Columbia between January 2003 and October 2007 [6]. Claims of REGARDS study participants in Medicare, a government health insurance program for US adults ≥ 65 years of age, were obtained from the Centers for Medicare and Medicaid Services Chronic Conditions Warehouse (CMS-CCW) [7]. Gout was not assessed at baseline in the REGARDS study. For this analysis, we included REGARDS study participants ≥ 65.5 years of age with continuous Medicare fee-for-service inpatient and outpatient coverage for ≥ 182 days prior to their baseline in-home study visit to identify gout through Medicare claims. After excluding participants with a history of HF, CHD, or stroke as defined in Supplemental Table 1, or without follow-up contacts for outcome identification, a total of 5713 REGARDS study participants were included in the current analysis (Supplemental Figure 1).

### Baseline assessment

REGARDS study participants completed a telephone interview and an in-home visit at baseline. During the in-home visit, trained health professionals conducted a physical examination, an electrocardiogram, and a medication inventory and collected blood and urine samples. Participants were requested to complete the Block 98 food frequency questionnaire (FFQ) and return it after the in-home visit using a pre-paid envelope.

For the current analysis, we used data on participants’ age, race, gender, geographic region of residence, income, education, alcohol consumption, smoking status, body mass index (BMI), physical activity, diabetes, chronic kidney disease (CKD), atrial fibrillation, systolic blood pressure, total cholesterol, high-density lipoprotein (HDL) cholesterol, C-reactive protein, and use of antihypertensive medication, diuretics, statin, aspirin, and cyclooxygenase-2-selective and non-cyclooxygenase-2-selective nonsteroidal anti-inflammatory drugs (NSAIDs), as defined in Supplemental Table 2. We also used scores for adherence to five dietary patterns (i.e., convenience, plant-based, sweets, southern and alcohol/salads) identified through factor analysis of Block 98 FFQ data [8]. The use of antigout medication, including allopurinol, colchicine, and probenecid, was defined based on the baseline medication inventory. Febuxostat was not approved by the Food and Drugs Administration until 2009 [9]. Gout was defined by ≥ 1 hospitalization or ≥ 2 outpatient visits with an International Classification of Diseases, ninth revision diagnosis code of 274.xx in Medicare claims at any time prior to each participant’s baseline examination [10, 11].

### Outcomes identification

REGARDS study participants or proxy respondents were followed through biannual telephone contacts to identify deaths and hospitalizations related with HF, CHD, and stroke events [6, 12]. Medical records for suspected events were retrieved, and hospitalizations for HF and myocardial infarctions (MI) were confirmed by trained clinicians following published guidelines [13–15]. Using hospital records from confirmed HF hospitalizations, the left ventricular ejection fraction (LVEF) was classified as reduced (LVEF < 50% or qualitative report of abnormal LVEF) or preserved (LVEF ≥ 50% or qualitative report of normal LVEF) [16]. Hospitalizations for stroke events were confirmed by a panel of neurologists following the World Health Organization definition [17]. Events characterized by symptoms lasting < 24 h with neuroimaging consistent with acute infarct or hemorrhage were also classified as strokes. When deaths were identified, trained clinicians determined whether a CHD or stroke event was the main underlying cause of death based on interviews with next-of-kin, medical records, death certificates, and autopsy reports [14, 18]. Data on incident HF hospitalizations, CHD (i.e., MI or CHD death), stroke (fatal or nonfatal), and all-cause mortality were available through December 31, 2016.

### Statistical analysis

We compared characteristics of REGARDS study participants with and without gout using chi-squared tests for categorical variables and *t* tests for continuous variables. We calculated the crude cumulative incidence by the Kaplan-Meier method and the incidence rate of HF hospitalization, CHD, stroke, and all-cause mortality among participants with and without gout, separately. Hazard ratios (HR) for HF hospitalization, CHD, stroke, and all-cause mortality, separately, comparing participants with versus without gout were calculated using four Cox regression models. Model 1 included adjustment for age, race, and gender. Model 2 included adjustment for age, race, gender, region of residence, income, and education. Model 3 included adjustment for variables in model 2 and alcohol consumption, current smoking, BMI, physical activity, and dietary patterns. Model 4 included adjustment for variables in model 3 and diabetes, CKD, atrial fibrillation, systolic blood pressure, total and HDL cholesterol, C-reactive protein, and use of antihypertensive medication, diuretics, statin, aspirin, and cyclooxygenase-2-selective and non-cyclooxygenase-2-selective NSAIDs. Also, we calculated the crude cumulative incidence, incidence rate, and HR for HF hospitalization with reduced and preserved LVEF, separately, among participants with and without gout.

To test whether HRs associated with gout differed by race or gender, we repeated the analysis using interaction terms between gout and race, and gout and gender, separately. Chained equations were used to obtain 35 multiple imputed datasets in Stata 13 (Stata Corp, College Station, TX) to retain REGARDS study participants with missing data in the regression models (Supplemental Table 3) [19].

### Sensitivity analyses

Few participants without a diagnosis of gout were taking antigout medication (*n* = 61). Antigout medications were not used to define gout in the main analysis as these may have other indications (e.g., inflammatory bowel disease). In a sensitivity analysis, we compared the risk for HF hospitalization, CHD, stroke, and all-cause mortality among participants with gout or taking antigout medication versus those without gout who were not taking antigout medication. Some participants had evidence of HF, CHD, or cerebrovascular disease based on Medicare claims as defined in Supplemental Table 4 before their baseline in-home study visit (*n* = 1096). We analyzed the risk for HF hospitalization, CHD, stroke, and all-cause mortality associated with gout after excluding these participants in a sensitivity analysis.

### Medicare cohort

We obtained claims data for a 5% random sample of Medicare beneficiaries from the CMS-CCW. For each beneficiary, we generated a random index date between January 1, 2008, and June 30, 2015. Beneficiaries ≥ 65.5 years of age with Medicare fee-for-service inpatient, outpatient, and pharmacy coverage for the preceding ≥ 182 days and without a history of HF, CHD, or cerebrovascular disease (as defined in Supplemental Table 4) on their index date were included in the analysis (*n* = 839,059, Supplemental Figure 2). We excluded Medicare beneficiaries before January 1, 2008, to allow for at least 182 days of inpatient, outpatient, and pharmacy coverage to define beneficiary characteristics as the Medicare pharmacy benefits program did not start until 2006 but its usage did not increase until 2007 [20]. Definitions of gout and beneficiary characteristics are provided in Supplemental Table 5.

Medicare beneficiaries were followed for HF, MI, and stroke hospitalizations and all-cause mortality as defined in Supplemental Table 6 from their index date through December 31, 2017, or loss of fee-for-service inpatient or outpatient coverage. We calculated the crude cumulative incidence and incidence rate of HF, MI, and stroke hospitalizations and all-cause mortality among beneficiaries with and without gout. We used three Cox regression models to calculate HRs for HF, MI, and stroke hospitalizations and all-cause mortality comparing beneficiaries with versus without gout. Model 1 included adjustment for age, race/ethnicity, and gender. Model 2 included adjustment for age, race/ethnicity, gender, region of residence, and Medicare-Medicaid eligible/low-income subsidy. Model 3 included adjustment for variables in model 2 and diabetes, CKD, atrial fibrillation, hypertension, and use of diuretics, statin (and statin intensity), and cyclooxygenase-2-selective and non-cyclooxygenase-2-selective NSAIDs. Data for some variables adjusted for in the analysis of the REGARDS study sample, including alcohol consumption, diet, smoking status, BMI, physical activity, blood pressure, serum cholesterol, and over-the-counter medications (e.g., aspirin), are not available in Medicare claims. We used a *Q* test for heterogeneity to compare fully adjusted HRs in the Medicare cohort versus those in the REGARDS study. We tested for interaction between gout and race/ethnicity, and gout and gender, separately, in the Medicare cohort as described for the REGARDS study.

## Results

Overall, 187 (3.3%) of 5713 REGARDS study participants included in the current analysis had gout. Compared to REGARDS study participants without gout, those with gout were older, and more likely to be black or men, have less than high school education, higher BMI or low physical activity, and have diet consistent with the southern dietary pattern, diabetes, CKD, or higher levels of systolic blood pressure or C-reactive protein (Table 1). Participants with gout had lower total and HDL cholesterol versus those without gout. A higher proportion of participants with versus without gout were taking antihypertensive medication and cyclooxygenase-2-selective NSAIDs.

**Table 1:** Baseline characteristics of REGARDS study participants included in the current analysis

Baseline characteristics	Participants without gout (*n* = 5526)	Participants with gout (*n* = 187)	*p* value
Age, years, mean (SD)	72.5 (5.6)	73.8 (6.1)	0.003
Black, *n* (%)	1711 (31.0)	83 (44.4)	< 0.001
Men, *n* (%)	2440 (44.2)	123 (65.8)	< 0.001
Region of residence, *n* (%)*			
Stroke belt	1995 (36.1)	61 (32.6)	
Stroke buckle	1258 (22.8)	50 (26.7)	0.39
Other US regions	2273 (41.1)	76 (40.6)	
< $25,000 annual income, *n* (%)	1790 (34.4)	57 (32.0)	0.51
Less than high school education, *n* (%)	727 (13.2)	38 (20.3)	0.005
Alcohol consumption, *n* (%)			
None	3527 (65.1)	117 (63.9)	
Moderate	1669 (30.8)	59 (32.2)	0.90
Heavy	225 (4.2)	7 (3.8)	
Current smoking, *n* (%)	485 (8.8)	12 (6.5)	0.26
Body mass index, *n* (%)			
< 25 kg/m^2^	1749 (31.8)	27 (14.6)	
25 to < 30 kg/m^2^	2182 (39.7)	67 (36.2)	< 0.001
≥ 30 kg/m^2^	1572 (28.6)	91 (49.2)	
Low physical activity, *n* (%)^†^	1794 (33.1)	74 (40.7)	0.03
Dietary patterns			
Convenience			
0 to < 25 percentile (least adherent)	1080 (25.2)	25 (19.4)	
25 to < 50 percentile	1074 (25.0)	31 (24.0)	0.19
50 to < 75 percentile	1074 (25.0)	31 (24.0)	
75 to 100 percentile (most adherent)	1063 (24.8)	42 (32.6)	
Plant-based			
0 to < 25 percentile (least adherent)	1070 (24.9)	35 (27.1)	
25 to < 50 percentile	1066 (24.8)	39 (30.2)	0.22
50 to < 75 percentile	1073 (25.0)	32 (24.8)	
75 to 100 percentile (most adherent)	1082 (25.2)	23 (17.8)	
Sweets			
0 to < 25 percentile (least adherent)	1062 (24.7)	43 (33.3)	
25 to < 50 percentile	1080 (25.2)	25 (19.4)	0.13
50 to < 75 percentile	1076 (25.1)	29 (22.5)	
75 to 100 percentile (most adherent)	1073 (25.0)	32 (24.8)	
Southern			
0 to < 25 percentile (least adherent)	1088 (25.4)	17 (13.2)	
25 to < 50 percentile	1082 (25.2)	23 (17.8)	< 0.001
50 to < 75 percentile	1068 (24.9)	37 (28.7)	
75 to 100 percentile (most adherent)	1053 (24.5)	52 (40.3)	
Alcohol and salads			
0 to < 25 percentile (least adherent)	1072 (25.0)	33 (25.6)	
25 to < 50 percentile	1073 (25.0)	32 (24.8)	0.79
50 to < 75 percentile	1077 (25.1)	28 (21.7)	
75 to 100 percentile (most adherent)	1069 (24.9)	36 (27.9)	
Diabetes, *n* (%)	903 (16.9)	52 (29.4)	< 0.001
Chronic kidney disease, *n* (%)	1266 (23.1)	85 (45.9)	< 0.001
Atrial fibrillation, *n* (%)	384 (7.1)	15 (8.3)	0.55
SBP, mm Hg, mean (SD)	129.5 (16.4)	132.6 (15.4)	0.01
Total cholesterol, mg/dL, mean (SD)	193.2 (38.2)	182.5 (39.5)	< 0.001
HDL cholesterol, mg/dL, mean (SD)	53.6 (16.6)	46.7 (17.2)	< 0.001
C-reactive protein > 3 mg/dL, *n* (%)	1862 (35.7)	94 (54.7)	< 0.001
Medication use, *n* (%)			
Antihypertensive medication	2764 (50.7)	132 (70.6)	< 0.001
Diuretics	1480 (26.8)	57 (30.5)	0.26
Statin	1590 (28.8)	55 (29.4)	0.85
Aspirin	2476 (44.8)	82 (43.9)	0.79
COX-2-selective NSAIDs	423 (7.7)	22 (11.8)	0.04
Non-COX-2-selective NSAIDs	828 (15.0)	30 (16.0)	0.69
Antigout medications^‡^			
Allopurinol	46 (0.8)	63 (33.7)	< 0.001
Colchicine	17 (0.3)	15 (8.0)	< 0.001
Probenecid	2 (0.0)	3 (1.6)	< 0.001

Definitions for baseline characteristics included in the analysis are provided in Supplemental Table 2

*COX* cyclooxygenase, *HDL* high-density lipoprotein, *NSAID* nonsteroidal anti-inflammatory drugs, *REGARDS* REasons for Geographic And Racial Differences in Stroke, *SBP* systolic blood pressure, *SD* standard deviation, *US* United States

*Stroke buckle includes coastal North Carolina, South Carolina, and Georgia. Stroke belt includes the remaining parts of North Carolina, South Carolina, and Georgia, and Tennessee, Mississippi, Alabama, Louisiana, and Arkansas. Other US regions include the remaining 40 contiguous US states and the District of Columbia

^†^Low physical activity is defined by self-reporting not engaging in any weekly activity intense enough to work up a sweat

^‡^The use of febuxostat was not analyzed at baseline in the REGARDS study as this medication was not approved until 2009 [9]

**Fig. 1:**
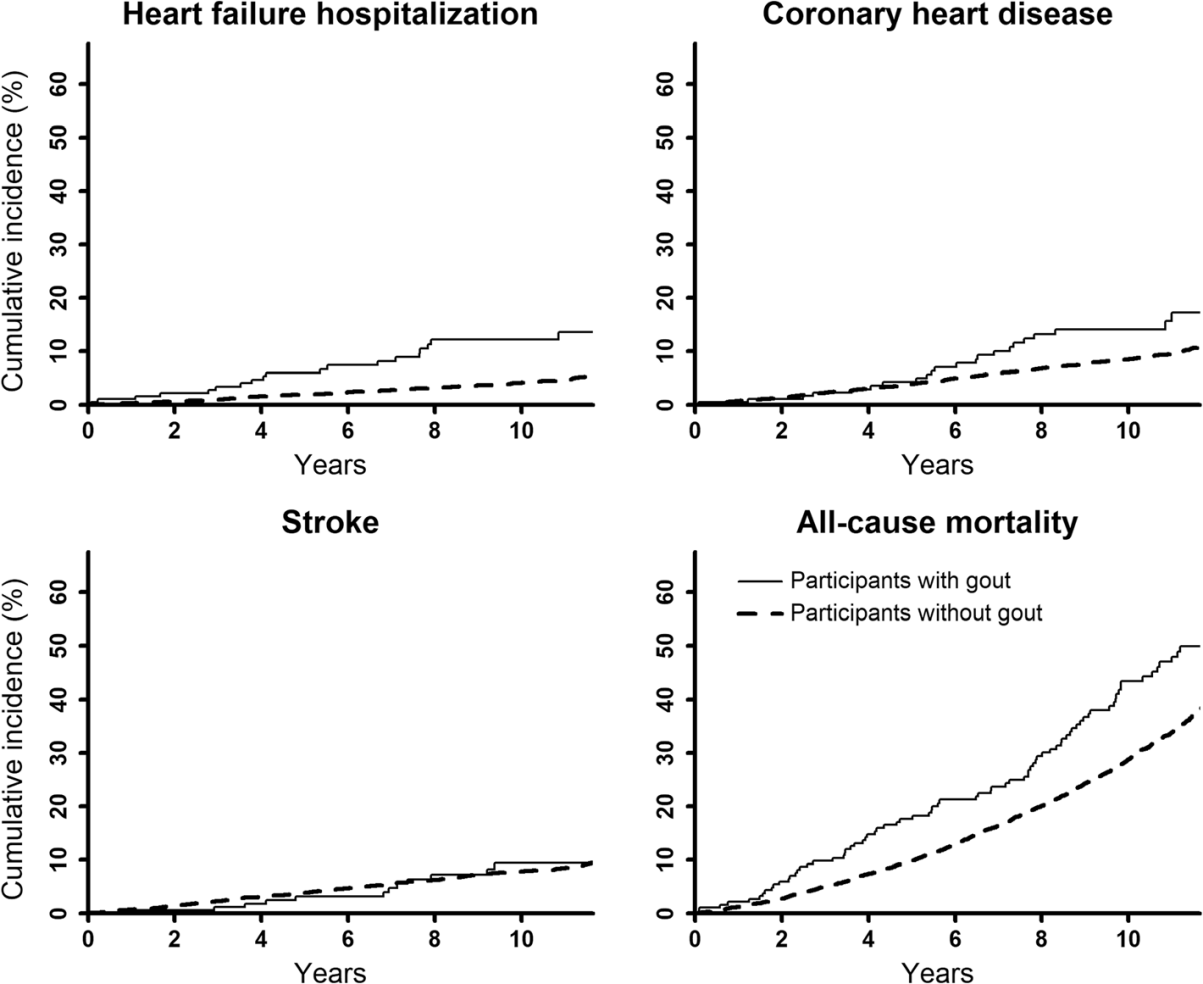
Cumulative incidence of HF hospitalization, CHD, stroke, and all-cause mortality among REGARDS study participants. CHD, coronary heart disease; HF, heart failure; REGARDS, REasons for Geographic And Racial Differences in Stroke. Cumulative incidence curves are unadjusted

REGARDS study participants with gout had higher cumulative incidence and incidence rate of HF hospitalization, CHD, and all-cause mortality, and similar cumulative incidence and incidence rate of stroke, versus their counterparts without gout (Fig. 1 and Table 2). After multivariable adjustment, gout was associated with a higher risk for HF hospitalization (HR 1.97, 95% confidence interval [95% CI] 1.22, 3.19). There were no statistically significant differences in the risk for CHD, stroke, and all-cause mortality among participants with versus without gout after multivariable adjustment. Gout was associated with an increased risk for HF hospitalization with both reduced and preserved LVEF (Supplemental Figure 3 and Table 3).

**Table 2:** Risk for HF hospitalization, coronary heart disease, stroke, and all-cause mortality among REGARDS study participants

	Participants without gout (*n* = 5526)	Participants with gout (*n* = 187)	*p* value
HF hospitalization			
Events/person-years	223/50,163	20/1522	
Incidence rate (95% CI)*	4.4 (3.9, 5.0)	13.1 (7.4, 18.9)	
Hazard ratio (95% CI)			
Model 1	1 (reference)	2.59 (1.63, 4.11)	< 0.001
Model 2	1 (reference)	2.59 (1.63, 4.11)	< 0.001
Model 3	1 (reference)	2.27 (1.41, 3.66)	0.001
Model 4	1 (reference)	1.97 (1.22, 3.19)	0.006
Coronary heart disease			
Events/person-years	453/48,904	24/1504	
Incidence rate (95% CI)*	9.3 (8.4, 10.1)	16.0 (9.6, 22.3)	
Hazard ratio (95% CI)			
Model 1	1 (reference)	1.47 (0.97, 2.22)	0.07
Model 2	1 (reference)	1.46 (0.97, 2.21)	0.07
Model 3	1 (reference)	1.38 (0.91, 2.09)	0.14
Model 4	1 (reference)	1.21 (0.79, 1.84)	0.39
Stroke			
Events/person-years	398/48,702	14/1512	
Incidence rate (95% CI)*	8.2 (7.4, 9.0)	9.3 (4.4, 14.1)	
Hazard ratio (95% CI)			
Model 1	1 (reference)	1.03 (0.60, 1.75)	0.93
Model 2	1 (reference)	1.01 (0.59, 1.73)	0.97
Model 3	1 (reference)	0.95 (0.56, 1.64)	0.87
Model 4	1 (reference)	0.83 (0.48, 1.43)	0.51
All-cause mortality			
Events/person-years	1858/50,142	85/1545	
Incidence rate (95% CI)*	37.1 (35.4, 38.7)	55.0 (43.3, 66.7)	
Hazard ratio (95% CI)			
Model 1	1 (reference)	1.24 (1.00, 1.54)	0.05
Model 2	1 (reference)	1.22 (0.98, 1.52)	0.07
Model 3	1 (reference)	1.19 (0.95, 1.48)	0.13
Model 4	1 (reference)	1.08 (0.86, 1.35)	0.51

Model 1 adjusts for age, race, and gender

Model 2 adjusts for age, race, gender, region of residence, income, and education

Model 3 adjusts for variables in model 2 plus alcohol consumption, current smoking, body mass index, physical activity, and dietary patterns

Model 4 adjusts for variables in model 3 plus diabetes, chronic kidney disease, atrial fibrillation, systolic blood pressure, total cholesterol, high-density lipoprotein cholesterol, C-reactive protein, and use of antihypertensive medication, diuretics, statin, aspirin, and cyclooxygenase-2-selective and non-cyclooxygenase-2-selective nonsteroidal anti-inflammatory drugs

The median (maximum) follow-up for all-cause mortality was 10.0 (13.9) years

*CI* confidence interval, *HF* heart failure, *REGARDS* REasons for Geographic And Racial Differences in Stroke

*Per 1000 person-years

**Table 3:** Risk for heart failure hospitalization with reduced and preserved LVEF among REGARDS study participants

	Participants without gout (*n* = 5526)	Participants with gout (*n* = 187)	*p* value
HF hospitalization with reduced LVEF			
Events/person-years	92/50,163	8/1522	
Incidence rate (95% CI)*	1.8 (1.5, 2.2)	5.3 (1.6, 8.9)	
Hazard ratio (95% CI)			
Model 1	1 (reference)	2.41 (1.16, 5.01)	0.02
Model 2	1 (reference)	2.49 (1.20, 5.17)	0.01
Model 3	1 (reference)	2.17 (1.03, 4.59)	0.04
Model 4	1 (reference)	1.77 (0.83, 3.79)	0.14
HF hospitalization with preserved LVEF			
Events/person-years	99/50,163	9/1522	
Incidence rate (95% CI)*	2.0 (1.6, 2.4)	5.9 (2.1, 9.8)	
Hazard ratio (95% CI)			
Model 1	1 (reference)	2.93 (1.46, 5.85)	< 0.001
Model 2	1 (reference)	2.85 (1.42, 5.70)	< 0.001
Model 3	1 (reference)	2.56 (1.25, 5.22)	0.01
Model 4	1 (reference)	2.32 (1.12, 4.79)	0.02

Model 1 adjusts for age, race, and gender

Model 2 adjusts for age, race, gender, region of residence, income, and education

Model 3 adjusts for variables in model 2 plus alcohol consumption, current smoking, body mass index, physical activity, and dietary patterns

Model 4 adjusts for variables in model 3 plus diabetes, chronic kidney disease, atrial fibrillation, systolic blood pressure, total cholesterol, high-density lipoprotein cholesterol, C-reactive protein, and use of antihypertensive medication, diuretics, statin, aspirin, and cyclooxygenase-2-selective and non-cyclooxygenase-2-selective nonsteroidal anti-inflammatory drugs

The LVEF could not be determined for 35 incident heart failure hospitalizations

*CI* confidence interval, *HF* heart failure, *LVEF* left ventricular ejection fraction, *REGARDS* REasons for Geographic And Racial Differences in Stroke

*Per 1000 person-years

The risk for HF hospitalization associated with gout was not statistically significantly different between black and white participants (Supplemental Table 7). Also, there was no evidence of an association of gout with CHD or stroke among black or white participants. Gout was associated with an increased risk for all-cause mortality among black participants, but not among white participants (multivariable-adjusted HRs [95% CI] 1.46 [1.05, 2.02] and 0.85 [0.61, 1.17], respectively, *p* value for the difference in HRs 0.02). When stratified by gender, gout was associated with an increased risk for HF hospitalization among both men and women, but there was no evidence of an association with CHD, stroke, or all-cause mortality in either group (Supplemental Table 8).

### Sensitivity analyses

REGARDS study participants with gout or taking antigout medication (*n* = 248; 4.3%) had a higher incidence rate of HF hospitalization, CHD, stroke, and all-cause mortality compared with their counterparts without gout who were not taking antigout medication (Supplemental Table 9). After multivariable adjustment, participants with gout or taking antigout medication had a higher risk for HF hospitalization versus those without gout who were not taking antigout medication, but there were no statistically significant differences in the risk for CHD, stroke, or all-cause mortality. After excluding 1096 REGARDS study participants with evidence of HF, CHD, or cerebrovascular disease based on Medicare claims, the fully adjusted HRs (95% CI) for HF hospitalization, CHD, stroke, and all-cause mortality associated with gout were 1.67 (0.90, 3.12), 1.01 (0.58, 1.76), 0.76 (0.38, 1.49), and 0.96 (0.72, 1.28), respectively (Supplemental Table 10).

### Medicare cohort

**Fig. 2:**
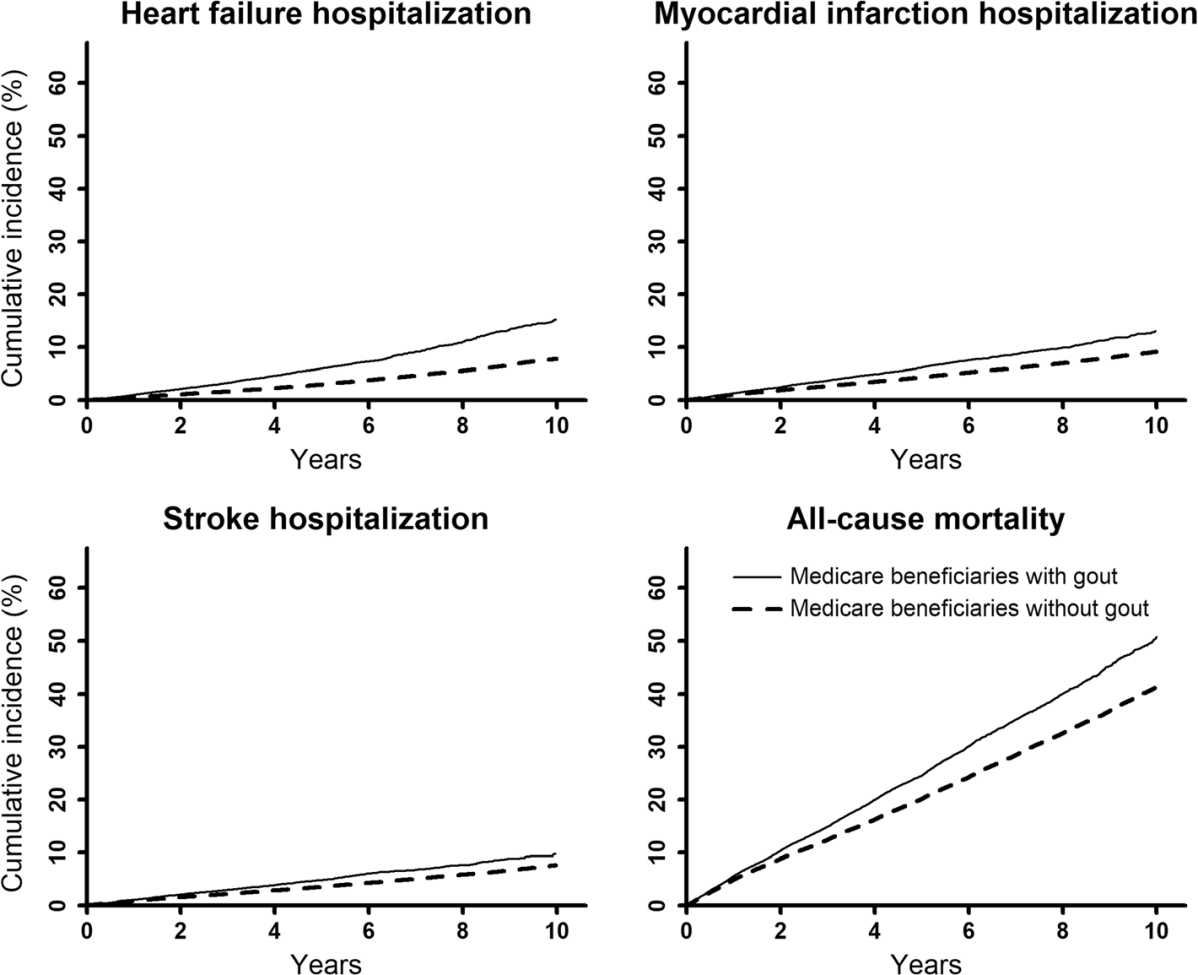
Cumulative incidence of HF, myocardial infarction, and stroke hospitalization and all-cause mortality among Medicare beneficiaries. HF, heart failure. Cumulative incidence curves are unadjusted

Medicare beneficiaries with gout (*n* = 29,753 [3.6%] of 839,059) were older and more likely to be non-Hispanic black or men; to have diabetes, CKD, atrial fibrillation, or hypertension; and to be taking a diuretic, statin, or non-cyclooxygenase-2-selective NSAID versus those without gout (Supplemental Table 11). Compared to Medicare beneficiaries without gout, those with gout had a higher cumulative incidence and incidence rate of HF, MI, and stroke hospitalizations and all-cause mortality (Fig. 2 and Table 4). After multivariable adjustment, Medicare beneficiaries with gout had a higher risk for HF hospitalization and a lower risk for all-cause mortality compared to those without gout. The multivariable-adjusted hazard ratio for MI hospitalization and stroke associated with gout was 1.06 (95% CI 1.00, 1.11) and 1.06 (95% CI 1.00, 1.12), respectively. Fully adjusted HRs in the Medicare cohort were not statistically significantly different versus those among REGARDS study participants (Table 5).

**Table 4:** Risk for heart failure, myocardial infarction, and stroke hospitalization and all-cause mortality among Medicare beneficiaries

	Medicare beneficiaries without gout (*n* = 809,306)	Medicare beneficiaries with gout (*n* = 29,753)	*p* value
Heart failure hospitalization			
Events/person-years	19,407/3,064,851	1477/115,128	–
Incidence rate (95% CI)*	6.3 (6.2, 6.4)	12.8 (12.2, 13.5)	–
Hazard ratio (95% CI)			
Model 1	1 (reference)	1.72 (1.63, 1.82)	< 0.001
Model 2	1 (reference)	1.74 (1.65, 1.83)	< 0.001
Model 3	1 (reference)	1.32 (1.25, 1.39)	< 0.001
Myocardial infarction hospitalization			
Events/person-years	27,553/3,039,727	1479/114,759	–
Incidence rate (95% CI)*	9.1 (9.0, 9.2)	12.9 (12.2, 13.5)	–
Hazard ratio (95% CI)			
Model 1	1 (reference)	1.19 (1.13, 1.26)	< 0.001
Model 2	1 (reference)	1.21 (1.15, 1.27)	< 0.001
Model 3	1 (reference)	1.06 (1.00, 1.11)	0.05
Stroke hospitalization			
Events/person-years	22,657/3,050,349	1152/115,356	–
Incidence rate (95% CI)*	7.4 (7.3, 7.5)	10.0 (9.4, 10.6)	–
Hazard ratio (95% CI)			
Model 1	1 (reference)	1.14 (1.07, 1.21)	< 0.001
Model 2	1 (reference)	1.14 (1.08, 1.22)	< 0.001
Model 3	1 (reference)	1.06 (1.00, 1.12)	0.07
All-cause mortality			
Events/person-years	145,623/3,101,396	7013/117,902	–
Incidence rate (95% CI)*	47.0 (46.7, 47.2)	59.5 (58.1, 60.9)	–
Hazard ratio (95% CI)			
Model 1	1 (reference)	1.02 (1.00, 1.05)	0.08
Model 2	1 (reference)	1.04 (1.02, 1.07)	0.001
Model 3	1 (reference)	0.95 (0.93, 0.98)	< 0.001

Model 1 includes adjustment for age, gender, and race/ethnicity

Model 2 includes adjustment for age, gender, race/ethnicity, region of residence, and Medicare-Medicaid eligible/low-income subsidy

Model 3 includes adjustment for variables in model 2 and diabetes, chronic kidney disease, atrial fibrillation, hypertension, and use of diuretics, statin (and statin intensity), and cyclooxygenase-2-selective and non-cyclooxygenase-2-selective nonsteroidal anti-inflammatory drugs

The median (maximum) follow-up for all-cause mortality was 3.1 (10.0) years

*CI* confidence interval

*Per 1000 person-years

**Table 5:** Fully adjusted hazard ratios for outcome events in the REGARDS study and Medicare cohort

	Hazard ratio (95% confidence interval)*		*Q* test
	REGARDS study (*n* = 5713)	Medicare cohort (*n* = 839,059)	*p* value
Heart failure	1.97 (1.22, 3.19)	1.32 (1.25, 1.39)	0.10
Coronary heart disease/myocardial infarction	1.21 (0.79, 1.84)	1.06 (1.00, 1.11)	0.55
Stroke	0.83 (0.48, 1.43)	1.06 (1.00, 1.12)	0.39
All-cause mortality	1.08 (0.86, 1.35)	0.95 (0.93, 0.98)	0.27

Hazard ratios in REGARDS include adjustment for age, race, gender, region of residence, income, education, alcohol consumption, current smoking, body mass index, physical activity, dietary patterns, diabetes, chronic kidney disease, atrial fibrillation, systolic blood pressure, total cholesterol, high-density lipoprotein cholesterol, C-reactive protein, and use of antihypertensive medication, diuretics, statin, aspirin, and cyclooxygenase-2-selective and non-cyclooxygenase-2-selective nonsteroidal anti-inflammatory drugs

Hazard ratios in Medicare include adjustment for age, gender, race/ethnicity, region of residence, Medicare-Medicaid eligible/low-income subsidy, diabetes, chronic kidney disease, atrial fibrillation, hypertension, and use of diuretics, statin (and statin intensity), and cyclooxygenase-2-selective and non-cyclooxygenase-2-selective nonsteroidal anti-inflammatory drugs

*Comparing older adults with versus without gout

HRs for HF, MI, and stroke hospitalizations and all-cause mortality associated with gout were not statistically significantly different across subgroups defined by race/ethnicity (Supplemental Table 12). After multivariable adjustment, gout was associated with a higher risk for HF and stroke hospitalization and lower all-cause mortality among men, and with a higher risk for HF and MI hospitalization among women (Supplemental Table 13).

## Discussion

In the current analysis, gout was associated with an increased risk for incident HF hospitalization among REGARDS study participants without a history of HF, CHD, or stroke. This association was present among blacks and whites and among men and women. In contrast, gout was not associated with incident CHD, stroke, or all-cause mortality after multivariable adjustment. The higher risk for incident HF associated with gout was also present in a large random sample of Medicare beneficiaries. These findings suggest that gout may be a risk factor for incident HF.

In the Framingham Offspring Study, a cohort composed exclusively of white adults, the HR for incident HF comparing participants with versus without gout was 1.74 (95% CI 1.03, 2.93) [21]. However, this study did not exclude individuals with a history of CHD or stroke at baseline and did not adjust for these conditions as part of the analysis. In the current analysis, gout was associated with a higher risk for incident HF in a cohort of individuals without a history of CHD or stroke and after adjustment for relevant risk factors for HF, including hypertension [22, 23], diabetes [23], cigarette smoking [23], obesity [24], high adherence to a southern dietary pattern [15], and use of NSAID [25]. These results support that the association of gout with incident HF is independent of a prior history of atherosclerosis disease or other well-known risk factors.

It has been previously shown that there is an attenuation in the association between many risk factors and cardiovascular events at an older age [26]. Prior studies suggest that gout is associated with a higher risk for CHD and stroke [2, 3]. However, this association may also be attenuated at an older age [2, 27]. In a population-based cohort of 704,503 adults with healthcare coverage through the National Health Insurance in Taiwan, multivariable-adjusted HRs for MI associated with gout were 1.59 (95% CI 1.12, 2.24) among those 20–44 years of age and 1.11 (95% CI 0.94, 1.32) among those ≥ 70 years of age [28]. In the current analysis of a random sample of Medicare beneficiaries ≥ 65.5 years of age, multivariable-adjusted HRs for MI and stroke were 1.06 (95% CI 1.00, 1.11) and 1.06 (95% CI 1.00, 1.12), respectively. Therefore, results from the current analysis do not exclude that gout may be associated with a small increase in the risk for CHD or stroke among older adults. Rather, results from the current study support that these associations may be attenuated at an older age. Given that the association of gout with incident CHD and stroke appears to be stronger among young versus older adults [2, 27, 28], it is possible that the association of gout with incident HF may also be stronger at a younger age.

Results from the current study suggest that a prior history of atherosclerotic cardiovascular disease or the development of atherosclerotic cardiovascular disease during follow-up do not explain the increased risk for HF associated with gout among older adults. However, the mechanisms behind this association remain unknown. In the current analysis, gout was associated with a higher risk for incident HF with preserved and reduced LVEF. HF with preserved and reduced LVEF has different underlying pathophysiology [29]. Patients with HF who have reduced LVEF are more likely to show upregulation of pathways related to cellular growth and metabolism, while those who have preserved LVEF are more likely to show upregulation of pathways related to inflammation and extracellular matrix reorganization [30]. Therefore, it is possible that gout may contribute to the development of HF through different pathways. Understanding the biological mechanisms involved in the development of HF in older adults with gout may contribute to identify therapeutic targets to prevent or treat HF in this population.

Inflammation, insulin resistance, and high serum urate levels may, in part, explain the increased risk for HF in adults with gout [31–36]. Interleukin-1 is a key inflammatory marker involved in the development of gout flares, as it was demonstrated in recent clinical trials with interleukin-1 blockade [37–39]. Interleukin-1 overexpression has been associated with the development of HF with preserved LVEF in animal models [31, 32]. Also, interleukin-1 blockade showed a dose-dependent reduction in the risk for HF hospitalization among adults with a previous MI and high C-reactive protein levels in the Canakinumab Anti-Inflammatory Thrombosis Outcomes Study (CANTOS) clinical trial [40]. Insulin resistance is common in adults with gout [41, 42]. Prior studies have shown that insulin resistance is associated with an increased risk for incident HF independently of diabetes and obesity [33, 34]. Furthermore, insulin resistance has been associated with both preserved and reduced LVEF HF [43]. Finally, hyperuricemia was associated with an increased risk for incident HF in the Cardiovascular Health Study (multivariable-adjusted HF 1.30, 95% CI 1.05, 1.60) [35]. Future studies should determine whether interleukin-1 blockade, pharmacologic insulin sensitization, or uric acid-lowering therapy is effective to reduce the risk for HF among adults with gout [44].

Gout was associated with a higher risk for all-cause mortality among middle-aged male participants without a history of CHD in the Health Professionals Follow-up Study (mean age 54 years) [45] and in the Multiple Risk Factor Intervention Trial (mean age 52 years) [46]. In the current analysis of REGARDS study participants ≥ 65.5 years of age without a history of CHD or stroke, gout was associated with a higher all-cause mortality among black but not among white participants. In the random sample of Medicare beneficiaries ≥ 65.5 years of age, gout was associated with a lower risk for all-cause mortality after multivariable adjustment, without evidence of effect modification by race or gender.

The current study has several strengths, including the use of data from the REGARDS study, a cohort with a rigorous cardiovascular event adjudication process. REGARDS study participants ≥ 65 years of age with Medicare fee-for-service coverage are representative of the general US population ≥ 65 years of age with the same healthcare insurance [7]. Also, we replicated the analysis in a random sample of Medicare beneficiaries. Despite these strengths, the current study has known and potential limitations. Although the use of claims to identify gout may result in some misclassification [47], diagnosis codes for gout have been shown to have a high sensitivity and specificity to identify gout-related visits in prior analyses [10, 48]. Also, results were consistent with the main analysis when we compared REGARDS study participants with gout or taking antigout medication versus their counterparts without gout who were not taking antigout medication. Many REGARDS study participants did not return complete Block 98 FFQ data [8], which required the use of multiple imputation to include dietary information in regression models. Levels of interleukin-1, insulin, and serum urate were not available for the current study.

## Conclusions

Results from the current study suggest that gout is a risk factor for incident HF among older adults. The higher risk for incident HF associated with gout was consistent for whites and blacks and men and women and was present in the REGARDS study cohort and in a random selection of Medicare beneficiaries. Interventions to reduce the excess risk for incident HF among older adults with gout are warranted.

## Supplementary information

**Additional file 1.**

## Data Availability

All data that support the findings of this study are included in the article or uploaded as supplementary information.

## Abbreviations

BMI: Body mass index
CHD: Coronary heart disease
CI: Confidence interval
CKD: Chronic kidney disease
CMS-CCW: Centers for Medicare and Medicaid Services Chronic Conditions Warehouse
FFQ: Food frequency questionnaire
HF: Heart failure
HR: Hazard ratio
LVEF: Left ventricular ejection fraction
MI: Myocardial infarction
NSAID: Nonsteroidal anti-inflammatory drug
REGARDS: REasons for Geographic And Racial Differences in Stroke
