# Supplementary material for: Gout is associated with an increased risk for incident heart failure among older adults: the REasons for Geographic And Racial Differences in Stroke (REGARDS) cohort study
Source: Arthritis Res Ther. 2020 Apr 16;22:86. doi: 10.1186/s13075-020-02175-2 (PMC7164141; doi:10.1186/s13075-020-02175-2)
Supplement: Supplementary file 1 — Additional file 1. [file 13075_2020_2175_MOESM1_ESM.docx]

**Gout is associated with an increased risk for incident heart failure among older adults: the REasons for Geographic And Racial Differences in Stroke (REGARDS) cohort study**

## SUPPLEMENTAL MATERIAL

Lisandro D. Colantonio,^1^ MD PhD, Kenneth G. Saag,^2^ MD MSc, Jasvinder A. Singh,^1,2,3^ MD MPH, Ligong Chen,^1^ PhD, Richard J. Reynolds,^2^ PhD, Angelo Gaffo,^2,3^ MD MSPH, Timothy B. Plante,^4^ MD MHS, Jeffrey R. Curtis,^1,2^ MD MS MPH, S. Louis Bridges, Jr.,^2^ MD PhD, Emily B. Levitan,^1^ ScD, Ninad S. Chaudhary,^1^ MBBS, MPH, George Howard,^5^ DrPH, Monika M. Safford,^6^ MD, Paul Muntner,^1^ PhD, Marguerite Ryan Irvin,^1^ PhD.

1. Department of Epidemiology, University of Alabama at Birmingham, Birmingham, AL, United States.
2. Division of Clinical Immunology and Rheumatology, University of Alabama at Birmingham, Birmingham, AL, United States.
3. Birmingham Veterans Affairs Medical Center, Birmingham, AL, United States.
4. Department of Medicine, Larner College of Medicine at the University of Vermont, Burlington, VT, United States.
5. Department of Biostatistics, University of Alabama at Birmingham, Birmingham, AL, United States.
6. Department of Medicine, Weill Cornell Medical College, New York, NY, United States.

Corresponding author: Lisandro D. Colantonio. 1720 2nd Ave South, RPHB 527C. Birmingham, AL 35294-0013. Phone: 205-259-6415. Fax: 205-975-7058. Email: [lcolantonio@uab.edu](mailto:lcolantonio@uab.edu).

**Supplemental Table 1.** Definitions for a history of heart failure, coronary heart disease and stroke in the REGARDS study.

| **Condition** | **Definition** |
| --- | --- |
| History of heart failure^1, 2^ | Use of one or more medications to treat heart failure based on the baseline REGARDS study medication inventory, including:   - Digoxin (if participants had no atrial fibrillation at baseline by self-report or based on the study electrocardiogram). - Carvedilol. - Spironolactone. - Hydralazine in combination with isosorbide mono- or di-nitrate. - Loop diuretic (i.e., furosemide, bumetanide, torsemide). - Angiotensin-converting enzyme inhibitor or angiotensin II receptor blocker in combination with a beta-blocker (if participants had systolic blood pressure <140 mm Hg, diastolic blood pressure <90 mm Hg and self-reported not taking medication to lower their blood pressure at baseline). |
| History of coronary heart disease^3, 4^ | A self-report of a prior diagnosis of myocardial infarction or a prior coronary revascularization during the baseline REGARDS study interview, or evidence of a previous myocardial infarction on the baseline study electrocardiogram. |
| History of stroke^3^ | A self-report of a prior diagnosis of stroke during the baseline REGARDS study interview. |
| REGARDS: REasons for Geographic And Racial Differences in Stroke. | |

**Supplemental Table 2.** Definitions for baseline characteristics of REGARDS study participants included in the current analysis.

| **Baseline characteristic** | **Definition** |
| --- | --- |
| Age | Calculated using date of birth provided by participants during their baseline computer-assisted telephone interview. |
| Gender and race | Based on gender and race self-reported by participants during their baseline computer-assisted telephone interview. |
| Region of residence | Based on the home address provided by participants during their baseline computer-assisted telephone interview and categorized as follows:   - Stroke buckle: includes coastal North Carolina, South Carolina and Georgia. - Stroke belt: includes the remaining parts of North Carolina, South Carolina and Georgia, and Tennessee, Mississippi, Alabama, Louisiana and Arkansas. - Other US regions: includes the remaining 40 contiguous US states and the District of Columbia. |
| Income | Based on the total household annual income from all sources that participants self-reported during their baseline computer-assisted telephone interview. |
| Education | Based on the highest education grade that participants reported have completed during their baseline computer-assisted telephone interview. |
| Alcohol consumption | Based on the number of drinks that participants self-reported having per week during their baseline computer-assisted telephone interview and categorized as follows:   - No alcohol consumption: 0 drinks per week. - Moderate alcohol consumption: >0 to 7 drinks per week for women and >0 to 14 drinks per week for men. - Heavy alcohol consumption: >7 drinks per week for women and >14 drinks per week for men. |
| Current smoking | Having smoked more than 100 cigarettes in lifetime and currently smoking cigarettes, even occasionally. |
| Body mass index | Calculated using body weight and height measured during the baseline in-home study examination. Specifically, body mass index was calculated as: body weight in kilograms / (height in meters)^2^. |
| Low physical activity | Self-reporting not engaging in any weekly activity intense enough to work up a sweat. |
| Diabetes | Fasting glucose ≥126 mg/dL, non-fasting glucose ≥200 mg/dL, or self-report of a prior diagnosis of diabetes with current use of insulin or oral hypoglycemic medication. |
| Chronic kidney disease | Self-report of being on dialysis, or a calculated estimated glomerular filtration rate <60 ml/min/1.73 m^2^ or albumin-to-creatinine ratio ≥30 mg/g. Estimated glomerular filtration rate was calculated using information on age, race, gender, and serum creatinine and a published equation from the Chronic Kidney Disease Epidemiology Collaboration.^5^ Using urine samples, albumin and creatinine were measured and used to calculate the albumin-to-creatinine ratio as: urinary albumin / urinary creatinine. |
| Atrial fibrillation | Presence of atrial fibrillation on the baseline study electrocardiogram or self-report of a prior diagnosis of atrial fibrillation during the baseline study interview. |
| Systolic blood pressure | Average of the two systolic blood pressure measurements during the baseline study examination. Blood pressure was measured by a trained health professional using the auscultatory method and an aneroid sphygmomanometer with an appropriately sized cuff. Prior to their first blood pressure measurement, participants rested for five minutes in a seated position with both feet on the floor. At least 30 seconds elapsed between each blood pressure measurement. |
| Total cholesterol | Measured by colorimetric reflectance spectrophotometry using blood samples collected during the baseline in-home examination. |
| High-density lipoprotein cholesterol | Measured by colorimetric reflectance spectrophotometry using blood samples collected during the baseline in-home examination. |
| C-reactive protein | Measured by particle enhanced immunonephelometry using blood samples collected during the baseline in-home examination. |
| Use of antihypertensive medications | Self-reporting taking medication to lower their blood pressure during the baseline computer-assisted telephone interview. |
| Use of diuretics | Having present any of the following medications in the baseline medication inventory: chlorothiazide, chlorthalidone, hydrochlorothiazide, indapamide, metolazone. Participants taking a loop diuretic (i.e., bumetanide, furosemide, torsemide) were defined as having a history of heart failure (see **Online Table 1**) and excluded from the analysis. |
| Use of statin | Having present any of the following medications in the baseline medication inventory: atorvastatin, fluvastatin, lovastatin, pitavastatin, pravastatin, rosuvastatin, simvastatin. |
| Use of aspirin | Self-reporting taking aspirin regularly during the baseline computer-assisted telephone interview. |
| Use of cyclooxygenase-2-selective NSAIDs | Having present any of the following medications in the baseline medication inventory: celecoxib, rofecoxib, valdecoxib. |
| Use of non-cyclooxygenase-2-selective NSAIDs | Having present any of the following medication in the baseline medication inventory: bromfenac, diclofenac, diflunisal, etodolac, fenoprofen, flurbiprofen, ibuprofen, indomethacin, ketoprofen, ketorolac, meclofenamate, mefenamic, meloxicam, nabumetone, naproxen, oxaprozin, piroxicam, salsalate, sulindac, tolmetin. |
| NSAIDs: nonsteroidal anti-inflammatory drugs; REGARDS: REasons for Geographic And Racial Differences in Stroke; US: United States. | |

**Supplemental Table 3.** Missing data among REGARDS study participants included in the current analysis.

| **Baseline characteristic** | **All participants**  **(n=5,713)** |
| --- | --- |
| Age | 0 (0.0) |
| Race | 0 (0.0) |
| Gender | 0 (0.0) |
| Region of residence | 0 (0.0) |
| Income | 333 (5.8) |
| Education | 1 (0.0) |
| Alcohol consumption | 109 (1.9) |
| Current smoking | 21 (0.4) |
| Body mass index | 25 (0.4) |
| Low physical activity | 107 (1.9) |
| Dietary patterns | 1,293 (22.6) |
| Diabetes | 208 (3.6) |
| Chronic kidney disease | 51 (0.9) |
| Atrial fibrillation | 134 (2.3) |
| Systolic blood pressure | 14 (0.2) |
| Total cholesterol | 207 (3.6) |
| HDL cholesterol | 246 (4.3) |
| C-reactive protein | 331 (5.8) |
| Antihypertensive medication | 78 (1.4) |
| Diuretics | 0 (0.0) |
| Statin | 0 (0.0) |
| Aspirin | 3 (0.1) |
| COX-2-selective NSAIDs | 0 (0.0) |
| Non-COX-2-selective NSAIDs | 0 (0.0) |
| Gout | 0 (0.0) |

COX: cyclooxygenase; HDL: high-density lipoprotein; NSAID: nonsteroidal anti-inflammatory drugs; REGARDS: REasons for Geographic And Racial Differences in Stroke.

Numbers in the table represent n (%).

**Supplemental Table 4.** Definitions for history of heart failure, coronary heart disease and cerebrovascular disease based on Medicare claims.

| **Exclusion criteria** | **Definition** |
| --- | --- |
| History of heart failure^6^ | Any of the following using all available claims prior to each beneficiary’s index date:*   - ≥1 hospitalization with a discharge diagnosis code of heart failure (ICD-9 diagnosis code of 402.01, 402.11, 402.91, 404.01, 404.03, 404.11, 404.13, 404.91, 404.93, 428.x) in any discharge diagnosis position - ≥2 physician evaluation and management outpatient visits with a diagnosis code of heart failure (ICD-9 diagnosis code of 402.01, 402.11, 402.91, 404.01, 404.03, 404.11, 404.13, 404.91, 404.93, 428.x) in any position on separate days. |
| History of CHD^7^ | Any of the following using all available claims prior to each beneficiary’s index date:*   - ≥1 hospitalization with an ICD-9 diagnosis code (any position) of 410.xx-414.xx, V45.81 or V45.82, or an ICD-9 procedure code of 00.66, 36.0, 36.01-36.19, 36.2. - ≥1 physician evaluation and management outpatient visit with an ICD-9 diagnosis code (any position) of 410.xx-414.xx, V45.81 or V45.82. - ≥1 outpatient claim or revenue center file or carrier line file with an ICD-9 procedure code of 00.66, 36.0, 36.01-36.19, 36.2 or CPT code of 33510-33519, 33521-33523, 33530, 33533-33536, 92980-92982, 92984, 92995, 92996, 92920, 92921, 92924, 92925, 92928, 92929, 92933, 92934, 92937, 92938, 92941, 92943, or 92944. |
| History of cerebrovascular disease^8^ | Any of the following using all available claims prior to each beneficiary’s index date:*   - ≥1 hospitalization with an ICD-9 diagnosis code (any position) of 430.xx, 431.xx, 433.10, 433.30, 433.x1, 434.x1 or 436.x, or an ICD-9 procedural code of 00.63, 38.42, 38.12, or 39.90. - ≥1 physician evaluation and management outpatient visit with an ICD-9 diagnosis code (any position) of 430.xx, 431.xx, 433.x1, 434.x1 or 436.x. - An inpatient or outpatient claims with a CPT code of 35301, 35390, 37215, 37216, 0005T, 0075T, or 0076. |
| CHD: coronary heart disease; CPT: current procedure terminology; ICD-9: International Classification of Diseases, ninth revision; REGARDS: REasons for Geographic And Racial Differences in Stroke.  * Among REGARDS study participants, the index date refers to the date of their baseline in-home study visit. For the 5% random sample of Medicare beneficiaries, the index date is a randomly generated date between January 1, 2008 and June 30, 2015. | |

**Supplemental Table 5.** Definitions of gout and other characteristics among Medicare beneficiaries included in the analysis.

| **Beneficiary characteristic** | **Definition** |
| --- | --- |
| Gout^9^ | Any of the following any time prior to each beneficiary’s index date:   - ≥1 hospitalization with a diagnosis code for gout (ICD-9 code 274.xx). - ≥2 outpatient visits with a diagnosis code for gout (ICD-9 code 274.xx) on separate days. |
| Age | Calculated on the index date using birthdates from Medicare beneficiary summary files. |
| Race/ethnicity | As defined in Medicare beneficiary summary files. |
| Gender | As defined in Medicare beneficiary summary files. |
| Region of residence | Based on the state of residence on each beneficiary’s index date obtained from Medicare beneficiary summary files.   - Northeast: includes Connecticut, Maine, Massachusetts, New Hampshire, New Jersey, New York, Pennsylvania, Rhode Island and Vermont. - North central: includes Illinois, Indiana, Iowa, Kansas, Michigan, Minnesota, Missouri, Nebraska, North Dakota, Ohio, South Dakota and Wisconsin - South: includes Alabama, Arkansas, Delaware, Florida, Georgia, Kentucky, Louisiana, Maryland, Mississippi, North Carolina, Oklahoma, South Carolina, Tennessee, Texas, Virginia, West Virginia and the District of Columbia. - West: includes Alaska, Arizona, California, Colorado, Hawaii, Idaho, Montana, Nevada, New Mexico, Oregon, Utah, Washington and Wyoming. |
| Medicare-Medicaid eligible/low-income subsidy | Receipt of any low-income subsidy under Medicare Part D or state reported dual eligible status code of 01-08 or state buy-in (value of ‘C’) from the entitlement variable for any month during the 182 days prior to each beneficiary’s index date, inclusive. |
| Diabetes^10, 11^ | Any of the following:   - ≥1 hospitalization with a discharge ICD-9 diagnosis code (any position) of 250.xx, 357.2, 362.0x, or 366.41 any time prior to each beneficiary’s index date, inclusive. - ≥2 physician evaluation and management outpatient visits with an ICD-9 diagnosis (any position) of 250.xx, 357.2, 362.0x, or 366.41, with the 2 visits occurring at least 7 days apart any time prior to each beneficiary’s index date, inclusive. - ≥1 pharmacy claim for an oral antidiabetic drug fill or insulin in the 182 days prior to each beneficiary’s index date, inclusive. |
| Chronic kidney disease^12, 13^ | Any of the following using all available claims prior to each beneficiary’s index date, inclusive:   - ≥1 hospitalization with a discharge diagnosis code of chronic kidney disease (ICD-9 diagnosis code of 016.0x, 095.4, 189.0, 189.9, 223.0, 236.91, 250.4x, 271.4, 274.1, 283.11, 403.x1, 403.x0, 404.x2, 404.x3, 404.x0, 404.x1, 440.1, 442.1, 447.3, 572.4, 580–588, 591, 642.1, 646.2, 753.12–753.17, 753.19, 753.2, 794.4) in any discharge diagnosis position. - ≥1 physician evaluation and management outpatient visit with a diagnosis code of chronic kidney disease in any position. - Having an indicator for end-stage renal disease in the beneficiary summary file. |
| Atrial fibrillation^14^ | Any of the following using all available claims prior to each beneficiary’s index date, inclusive:   - ≥1 hospitalization with an ICD-9 discharge diagnosis code (any position) of 427.31 in any discharge diagnosis position. - ≥2 physician evaluation and management outpatient visits with ICD-9 diagnosis (any position) of 427.31 on separate days. |
| Hypertension^15^ | Any of the following:   - ≥1 hospitalization with an ICD-9 discharge diagnosis code of 401.x, 403.0x, 403.1x, 403.9x in any discharge diagnosis position any time prior to each beneficiary’s index date, inclusive. - ≥2 physician evaluation and management outpatient visits with an ICD-9 diagnosis code of 401.x, 403.0x, 403.1x, 403.9x in any position, at least 30 days apart any time prior to each beneficiary’s index date, inclusive. - At least 1 pharmacy claim for an antihypertensive medication in the 182 days prior to each beneficiary’s index date, inclusive. |
| Use of diuretics | Having a pharmacy fill for any of the following medications in the 182 days prior to each beneficiary’s index date, inclusive: bumetanide, chlorothiazide, chlorthalidone, furosemide, hydrochlorothiazide, indapamide, metolazone, and torsemide. |
| Use of statins and intensity | Having a pharmacy fill for any of the following medications in the 182 days prior to each beneficiary’s index date, inclusive: atorvastatin, fluvastatin, lovastatin, pitavastatin, pravastatin, rosuvastatin and simvastatin. High-intensity statin includes atorvastatin 40-80 mg/day, rosuvastatin 20-40 mg/day and simvastatin 80 mg/day. All other statin types and dosages were defined as low/moderate-intensity. |
| Use of cyclooxygenase-2-selective NSAIDs | Having a pharmacy fill for any of the following medications in the 182 days prior to each beneficiary’s index date, inclusive: celecoxib, rofecoxib and valdecoxib. |
| Use of non-cyclooxygenase-2-selective NSAIDs | Having a pharmacy fill for any of the following medications in the 182 days prior to each beneficiary’s index date, inclusive: bromfenac, diclofenac, diflunisal, etodolac, fenoprofen, flurbiprofen, ibuprofen, indomethacin, ketoprofen, ketorolac, meclofenamate, mefenamic, meloxicam, nabumetone, naproxen, oxaprozin, piroxicam, salsalate, sulindac, and tolmetin. |
| Use of allopurinol | Having a pharmacy fill for allopurinol in the 182 days prior to each beneficiary’s index date, inclusive. |
| Use of colchicine | Having a pharmacy fill for colchicine in the 182 days prior to each beneficiary’s index date, inclusive. |
| Use of probenecid | Having a pharmacy fill for probenecid in the 182 days prior to each beneficiary’s index date, inclusive. |
| Use of febuxostat | Having a pharmacy fill for febuxostat in the 182 days prior to each beneficiary’s index date, inclusive. |
| ICD-9: International Classification of Diseases, ninth revision; NSAID: nonsteroidal anti-inflammatory drugs. | |

**Supplemental Table 6**. Definitions for heart failure, myocardial infarction and stroke hospitalizations, and all-cause mortality among Medicare beneficiaries included in the analysis.

| **Outcome** | **Definition** |
| --- | --- |
| Heart failure hospitalization^6^ | A hospitalization with a discharge diagnosis code for heart failure (i.e., an ICD-9 diagnosis code of 402.01, 402.11, 402.91, 404.01, 404.03, 404.11, 404.13, 404.91, 404.93, 428.x, or an ICD-10 diagnosis code of I11.0, I13.0, I13.2, I50.1, I50.20, I50.21, I50.22, I50.23, I50.30, I50.31, I50.32, I50.33, I50.40, I50.41, I50.42, I50.43, I50.9) in the primary discharge diagnosis position. |
| Myocardial infarction hospitalization^16^ | An overnight hospitalization with a discharge diagnosis code for acute myocardial infarction (i.e., an ICD-9 diagnosis code of 410.xx, except 410.x2, which represent a subsequent episode of care, or an ICD-10 diagnosis code of code I21.xx) recorded in any discharge diagnosis position. |
| Stroke hospitalization^8^ | A hospitalization with an ICD-9 discharge diagnosis code of 430.xx, 431.xx, 433.x1, 434.x1 or 436.xx or an ICD-10 discharge diagnosis code of I60, I61 or I63 in the primary discharge diagnosis position. |
| All-cause mortality | Based on death dates in Medicare beneficiary summary files. |
| ICD-9: International Classification of Diseases, ninth revision; ICD-10: International Classification of Diseases, tenth revision.  Medicare switched from using ICD-9 to ICD-10 codes in October 2015.^17^ | |

**Supplemental Table 7.** Incidence rates and hazard ratios for heart failure hospitalization, coronary heart disease, stroke and all-cause mortality among REGARDS study participants with versus without gout stratified by race.

|  | **Black participants** | | **White participants** | |  |
| --- | --- | --- | --- | --- | --- |
|  | **without gout**  **(n=1,711)** | **with gout**  **(n=83)** | **without gout**  **(n=3,815)** | **with gout**  **(n=104)** | **p-value†** |
| **Heart failure hospitalization** |  |  |  |  |  |
| Events / person-years | 68 / 14,990 | 8 / 631 | 155 / 35,173 | 12 / 891 |  |
| Incidence rate (95% CI)* | 4.5 (3.5, 5.6) | 12.7 (3.9, 21.5) | 4.4 (3.7, 5.1) | 13.5 (5.8, 21.1) |  |
| Hazard ratio (95% CI) |  |  |  |  |  |
| Model 1 | 1 (reference) | 2.78 (1.33, 5.81) | 1 (reference) | 2.43 (1.34, 4.40) | 0.88 |
| Model 2 | 1 (reference) | 2.57 (1.22, 5.42) | 1 (reference) | 2.57 (1.41, 4.66) | 0.93 |
| Model 3 | 1 (reference) | 1.96 (0.90, 4.29) | 1 (reference) | 2.44 (1.33, 4.50) | 0.84 |
| Model 4 | 1 (reference) | 1.74 (0.77, 3.92) | 1 (reference) | 2.15 (1.16, 4.01) | 0.91 |
| **Coronary heart disease** |  |  |  |  |  |
| Events / person-years | 123 / 14,662 | 10 / 655 | 330 / 34,242 | 14 / 849 |  |
| Incidence rate (95% CI)* | 8.4 (6.9, 9.9) | 15.3 (5.8, 24.7) | 9.6 (8.6, 10.7) | 16.5 (7.8, 25.1) |  |
| Hazard ratio (95% CI) |  |  |  |  |  |
| Model 1 | 1 (reference) | 1.69 (0.88, 3.22) | 1 (reference) | 1.34 (0.78, 2.30) | 0.62 |
| Model 2 | 1 (reference) | 1.52 (0.79, 2.93) | 1 (reference) | 1.39 (0.81, 2.38) | 0.84 |
| Model 3 | 1 (reference) | 1.46 (0.74, 2.88) | 1 (reference) | 1.34 (0.78, 2.32) | 0.77 |
| Model 4 | 1 (reference) | 1.33 (0.66, 2.67) | 1 (reference) | 1.11 (0.64, 1.94) | 0.58 |
| **Stroke** |  |  |  |  |  |
| Events / person-years | 127 / 14,432 | 0 / 660 | 271 / 34,271 | 13 / 852 |  |
| Incidence rate (95% CI)* | 8.8 (7.3, 10.3) | 0.0 (0.0, 4.5) | 7.9 (7.0, 8.8) | 15.3 (7.0, 23.6) |  |
| Hazard ratio (95% CI) |  |  |  |  |  |
| Model 1 | 1 (reference) | ‡ | 1 (reference) | 1.62 (0.92, 2.84) | - |
| Model 2 | 1 (reference) | ‡ | 1 (reference) | 1.63 (0.93, 2.86) | - |
| Model 3 | 1 (reference) | ‡ | 1 (reference) | 1.66 (0.94, 2.94) | - |
| Model 4 | 1 (reference) | ‡ | 1 (reference) | 1.37 (0.77, 2.44) | - |
| **All-cause mortality** |  |  |  |  |  |
| Events / person-years | 579 / 14,935 | 45 / 661 | 1,279 / 35,207 | 40 / 884 |  |
| Incidence rate (95% CI)* | 38.8 (35.6, 41.9) | 68.1 (48.2, 88.0) | 36.3 (34.3, 38.3) | 45.2 (31.2, 59.3) |  |
| Hazard ratio (95% CI) |  |  |  |  |  |
| Model 1 | 1 (reference) | 1.66 (1.22, 2.25) | 1 (reference) | 0.97 (0.70, 1.32) | 0.01 |
| Model 2 | 1 (reference) | 1.56 (1.15, 2.13) | 1 (reference) | 1.00 (0.73, 1.37) | 0.04 |
| Model 3 | 1 (reference) | 1.52 (1.11, 2.10) | 1 (reference) | 0.98 (0.71, 1.35) | 0.06 |
| Model 4 | 1 (reference) | 1.46 (1.05, 2.02) | 1 (reference) | 0.85 (0.61, 1.17) | 0.02 |
| CI: confidence interval; REGARDS: REasons for Geographic And Racial Differences in Stroke.  * Per 1,000 person-years.  † Comparing hazard ratios associated with gout among black and white participants.  ‡ Hazard ratios were not calculated as there were no stroke events among black participants with gout.  Model 1 adjusts for age and gender.  Model 2 adjusts for age, gender, region of residence, income and education.  Model 3 adjusts for variables in Model 2 plus alcohol consumption, current smoking, body mass index, physical activity and dietary patterns.  Model 4 adjusts for variables in Model 3 plus diabetes, chronic kidney disease, atrial fibrillation, systolic blood pressure, total cholesterol, high-density lipoprotein cholesterol, C-reactive protein, and use of antihypertensive medication, diuretics, statin, aspirin, and cyclooxygenase-2-selective and non-cyclooxygenase-2-selective nonsteroidal anti-inflammatory drugs. | | | | | |

**Supplemental Table 8.** Incidence rates and hazard ratios for heart failure hospitalization, coronary heart disease, stroke and all-cause mortality among REGARDS study participants with versus without gout stratified by gender.

|  | **Men** | | **Women** | |  |
| --- | --- | --- | --- | --- | --- |
|  | **without gout**  **(n=2,440)** | **with gout**  **(n=123)** | **without gout**  **(n=3,086)** | **with gout**  **(n=64)** | **p-value†** |
| **Heart failure hospitalization** |  |  |  |  |  |
| Events / person-years | 110 / 22,437 | 12 / 1,058 | 113 / 27,726 | 8 / 463 |  |
| Incidence rate (95% CI)* | 4.9 (4.0, 5.8) | 11.3 (4.9, 17.8) | 4.1 (3.3, 4.8) | 17.3 (5.3, 29.2) |  |
| Hazard ratio (95% CI) |  |  |  |  |  |
| Model 1 | 1 (reference) | 2.20 (1.21, 3.99) | 1 (reference) | 3.55 (1.71, 7.37) | 0.35 |
| Model 2 | 1 (reference) | 2.25 (1.24, 4.10) | 1 (reference) | 3.25 (1.55, 6.80) | 0.48 |
| Model 3 | 1 (reference) | 2.17 (1.17, 4.03) | 1 (reference) | 2.83 (1.31, 6.09) | 0.43 |
| Model 4 | 1 (reference) | 2.07 (1.10, 3.90) | 1 (reference) | 2.34 (1.06, 5.17) | 0.62 |
| **Coronary heart disease** |  |  |  |  |  |
| Events / person-years | 257 / 21,742 | 18 / 1,025 | 196 / 27,162 | 6 / 480 |  |
| Incidence rate (95% CI)* | 11.8 (10.4, 13.3) | 17.6 (9.5, 25.7) | 7.2 (6.2, 8.2) | 12.5 (2.5, 22.5) |  |
| Hazard ratio (95% CI) |  |  |  |  |  |
| Model 1 | 1 (reference) | 1.46 (0.90, 2.36) | 1 (reference) | 1.50 (0.66, 3.41) | 0.94 |
| Model 2 | 1 (reference) | 1.48 (0.92, 2.39) | 1 (reference) | 1.34 (0.59, 3.06) | 0.91 |
| Model 3 | 1 (reference) | 1.41 (0.87, 2.30) | 1 (reference) | 1.23 (0.53, 2.84) | 0.88 |
| Model 4 | 1 (reference) | 1.25 (0.76, 2.05) | 1 (reference) | 1.05 (0.45, 2.44) | 0.77 |
| **Stroke** |  |  |  |  |  |
| Events / person-years | 188 / 21,827 | 12 / 1,024 | 210 / 26,876 | 2 / 488 |  |
| Incidence rate (95% CI)* | 8.6 (7.4, 9.8) | 11.7 (5.1, 18.3) | 7.8 (6.8, 8.9) | 4.1 (0.0, 9.8) |  |
| Hazard ratio (95% CI) |  |  |  |  |  |
| Model 1 | 1 (reference) | 1.34 (0.75, 2.40) | 1 (reference) | ‡ | - |
| Model 2 | 1 (reference) | 1.37 (0.76, 2.46) | 1 (reference) | ‡ | - |
| Model 3 | 1 (reference) | 1.34 (0.74, 2.44) | 1 (reference) | ‡ | - |
| Model 4 | 1 (reference) | 1.23 (0.67, 2.24) | 1 (reference) | ‡ | - |
| **All-cause mortality** |  |  |  |  |  |
| Events / person-years | 973 / 22,482 | 54 / 1,052 | 885 / 27,660 | 31 / 493 |  |
| Incidence rate (95% CI)* | 43.3 (40.6, 46.0) | 51.3 (37.6, 65.0) | 32.0 (29.9, 34.1) | 62.9 (40.8, 85.1) |  |
| Hazard ratio (95% CI) |  |  |  |  |  |
| Model 1 | 1 (reference) | 1.11 (0.85, 1.46) | 1 (reference) | 1.56 (1.09, 2.25) | 0.18 |
| Model 2 | 1 (reference) | 1.15 (0.87, 1.52) | 1 (reference) | 1.42 (0.99, 2.05) | 0.44 |
| Model 3 | 1 (reference) | 1.14 (0.86, 1.51) | 1 (reference) | 1.34 (0.92, 1.94) | 0.59 |
| Model 4 | 1 (reference) | 1.11 (0.83, 1.47) | 1 (reference) | 1.10 (0.76, 1.61) | 0.78 |
| CI: confidence interval; REGARDS: REasons for Geographic And Racial Differences in Stroke.  * Per 1,000 person-years.  † Comparing hazard ratios associated with gout among men and women.  ‡ Hazard ratios were not calculated as there were few stroke events among women with gout.  Model 1 adjusts for age and race.  Model 2 adjusts for age, race, region of residence, income and education.  Model 3 adjusts for variables in Model 2 plus alcohol consumption, current smoking, body mass index, physical activity and dietary patterns.  Model 4 adjusts for variables in Model 3 plus diabetes, chronic kidney disease, atrial fibrillation, systolic blood pressure, total cholesterol, high-density lipoprotein cholesterol, C-reactive protein, and use of antihypertensive medication, diuretics, statin, aspirin, and cyclooxygenase-2-selective and non-cyclooxygenase-2-selective nonsteroidal anti-inflammatory drugs. | | | | | |

**Supplemental Table 9.** Incidence rates and hazard ratios for heart failure hospitalization, coronary heart disease, stroke and all-cause mortality among REGARDS study participants with gout or taking antigout medication versus those without gout who were not taking antigout medication.

|  | **Participants without gout and not taking antigout medication***  **(n=5,465)** | **Participants with gout or taking antigout medication***  **(n=248)** | **P-value** |
| --- | --- | --- | --- |
| **Heart failure hospitalization** |  |  |  |
| Events / person-years | 214 / 49,641 | 29 / 2,044 |  |
| Incidence rate (95% CI)† | 4.3 (3.7, 4.9) | 14.2 (9.0, 19.4) |  |
| Hazard ratio (95% CI) |  |  |  |
| Model 1 | 1 (reference) | 2.96 (1.99, 4.39) | <0.001 |
| Model 2 | 1 (reference) | 2.99 (2.01, 4.43) | <0.001 |
| Model 3 | 1 (reference) | 2.69 (1.79, 4.05) | <0.001 |
| Model 4 | 1 (reference) | 2.22 (1.46, 3.37) | <0.001 |
| **Coronary heart disease** |  |  |  |
| Events / person-years | 440 / 48,383 | 37 / 2,025 |  |
| Incidence rate (95% CI)† | 9.1 (8.2, 9.9) | 18.3 (12.4, 24.2) |  |
| Hazard ratio (95% CI) |  |  |  |
| Model 1 | 1 (reference) | 1.72 (1.23, 2.42) | 0.002 |
| Model 2 | 1 (reference) | 1.73 (1.23, 2.43) | 0.001 |
| Model 3 | 1 (reference) | 1.63 (1.16, 2.31) | 0.005 |
| Model 4 | 1 (reference) | 1.40 (0.99, 1.99) | 0.06 |
| **Stroke** |  |  |  |
| Events / person-years | 392 / 48,183 | 20 / 2,031 |  |
| Incidence rate (95% CI)† | 8.1 (7.3, 8.9) | 9.8 (5.5, 14.2) |  |
| Hazard ratio (95% CI) |  |  |  |
| Model 1 | 1 (reference) | 1.11 (0.70, 1.74) | 0.66 |
| Model 2 | 1 (reference) | 1.10 (0.70, 1.73) | 0.67 |
| Model 3 | 1 (reference) | 1.04 (0.66, 1.65) | 0.87 |
| Model 4 | 1 (reference) | 0.89 (0.56, 1.42) | 0.62 |
| **All-cause mortality** |  |  |  |
| Events / person-years | 1,830 / 49,604 | 113 / 2,083 |  |
| Incidence rate (95% CI)† | 36.9 (35.2, 38.6) | 54.2 (44.2, 64.2) |  |
| Hazard ratio (95% CI) |  |  |  |
| Model 1 | 1 (reference) | 1.25 (1.03, 1.51) | 0.02 |
| Model 2 | 1 (reference) | 1.25 (1.03, 1.51) | 0.02 |
| Model 3 | 1 (reference) | 1.22 (1.01, 1.49) | 0.04 |
| Model 4 | 1 (reference) | 1.08 (0.88, 1.31) | 0.47 |
| CI: confidence interval; REGARDS: REasons for Geographic And Racial Differences in Stroke.  * Antigout medications include allopurinol, probenecid and colchicine. Febuxostat was not analyzed at baseline in the REGARDS study as this medication was not approved until 2009.^18^  † Per 1,000 person-years.  Model 1 adjusts for age, race and gender.  Model 2 adjusts for age, race, gender, region of residence, income and education.  Model 3 adjusts for variables in Model 2 plus alcohol consumption, current smoking, body mass index, physical activity and dietary patterns.  Model 4 adjusts for variables in Model 3 plus diabetes, chronic kidney disease, atrial fibrillation, systolic blood pressure, total cholesterol, high-density lipoprotein cholesterol, C-reactive protein, and use of antihypertensive medication, diuretics, statin, aspirin, and cyclooxygenase-2-selective and non-cyclooxygenase-2-selective nonsteroidal anti-inflammatory drugs. | | | |

**Supplemental Table 10.** Risk for HF hospitalization, CHD, stroke, and all-cause mortality among REGARDS study participants. Sensitivity analysis excluding REGARDS study participants meeting the definition of a history of CHD, stroke or HF using Medicare claims before their baseline in-home study visit.

|  | **Participants without gout**  **(n=4,485)** | **Participants with gout**  **(n=132)** | **p-value** |
| --- | --- | --- | --- |
| **HF hospitalization** |  |  |  |
| Events / person-years | 162 / 41,581 | 12 / 1,168 |  |
| Incidence rate (95% CI)* | 3.9 (3.3, 4.5) | 10.3 (4.5, 16.1) |  |
| Hazard ratio (95% CI) |  |  |  |
| Model 1 | 1 (reference) | 2.19 (1.21, 3.95) | 0.01 |
| Model 2 | 1 (reference) | 2.19 (1.21, 3.97) | 0.01 |
| Model 3 | 1 (reference) | 1.95 (1.06, 3.58) | 0.03 |
| Model 4 | 1 (reference) | 1.67 (0.90, 3.12) | 0.11 |
| **Coronary heart disease** |  |  |  |
| Events / person-years | 343 / 40,584 | 14 / 1,138 |  |
| Incidence rate (95% CI)* | 8.5 (7.6, 9.3) | 12.3 (5.9, 18.7) |  |
| Hazard ratio (95% CI) |  |  |  |
| Model 1 | 1 (reference) | 1.22 (0.71, 2.09) | 0.46 |
| Model 2 | 1 (reference) | 1.21 (0.71, 2.07) | 0.49 |
| Model 3 | 1 (reference) | 1.17 (0.68, 2.03) | 0.56 |
| Model 4 | 1 (reference) | 1.01 (0.58, 1.76) | 0.96 |
| **Stroke** |  |  |  |
| Events / person-years | 287 / 40,502 | 9 / 1,131 |  |
| Incidence rate (95% CI)* | 7.1 (6.3, 7.9) | 8.0 (2.8, 13.2) |  |
| Hazard ratio (95% CI) |  |  |  |
| Model 1 | 1 (reference) | 0.97 (0.50, 1.89) | 0.92 |
| Model 2 | 1 (reference) | 0.94 (0.48, 1.84) | 0.87 |
| Model 3 | 1 (reference) | 0.87 (0.44, 1.70) | 0.68 |
| Model 4 | 1 (reference) | 0.76 (0.38, 1.49) | 0.42 |
| **All-cause mortality** |  |  |  |
| Events / person-years | 1,345 / 41,533 | 52 / 1,159 |  |
| Incidence rate (95% CI)* | 32.4 (30.7, 34.1) | 44.9 (32.7, 57.1) |  |
| Hazard ratio (95% CI) |  |  |  |
| Model 1 | 1 (reference) | 1.10 (0.84, 1.46) | 0.49 |
| Model 2 | 1 (reference) | 1.07 (0.81, 1.42) | 0.62 |
| Model 3 | 1 (reference) | 1.03 (0.78, 1.37) | 0.82 |
| Model 4 | 1 (reference) | 0.96 (0.72, 1.28) | 0.79 |
| CHD: coronary heart disease; CI: confidence interval; HF: heart failure; REGARDS: REasons for Geographic And Racial Differences in Stroke.  * Per 1,000 person-years.  Definitions of a history of CHD, stroke or HF using Medicare claims are provided in **Supplemental Table 4**.  Model 1 adjusts for age, race and gender.  Model 2 adjusts for age, race, gender, region of residence, income and education.  Model 3 adjusts for variables in Model 2 plus alcohol consumption, current smoking, body mass index, physical activity and dietary patterns.  Model 4 adjusts for variables in Model 3 plus diabetes, chronic kidney disease, atrial fibrillation, systolic blood pressure, total cholesterol, high-density lipoprotein cholesterol, C-reactive protein, and use of antihypertensive medication, diuretics, statin, aspirin, and cyclooxygenase-2-selective and non-cyclooxygenase-2-selective nonsteroidal anti-inflammatory drugs. | | | |

**Supplemental Table 11.** Baseline characteristics of Medicare beneficiaries included in the analysis.

|  | **Medicare beneficiaries without gout**  **(n=809,306)** | **Medicare beneficiaries with gout**  **(n=29,753)** |
| --- | --- | --- |
| Calendar year,* n (%) |  |  |
| 2008 | 116,295 (14.4) | 4,197 (14.1) |
| 2009 | 80,208 (9.9) | 3,012 (10.1) |
| 2010 | 70,450 (8.7) | 2,657 (8.9) |
| 2011 | 70,519 (8.7) | 2,736 (9.2) |
| 2012 | 76,118 (9.4) | 2,987 (10.0) |
| 2013 | 107,215 (13.3) | 4,279 (14.4) |
| 2014 | 128,238 (15.9) | 4,739 (15.9) |
| 2015 | 160,263 (19.8) | 5,146 (17.3) |
| Age, years, mean (SD) | 73.4 (7.5) | 75.4 (7.5) |
| Age, years, n (%) |  |  |
| 65.5-70 | 366,706 (45.3) | 8,907 (29.9) |
| 71-75 | 174,439 (21.6) | 7,361 (24.7) |
| 76-80 | 111,147 (13.7) | 5,635 (18.9) |
| 81-85 | 78,336 (9.7) | 4,122 (13.9) |
| ≥86 | 78,678 (9.7) | 3,728 (12.5) |
| Race, n (%) |  |  |
| Non-Hispanic white | 683,937 (84.5) | 23,482 (78.9) |
| Non-Hispanic black | 63,255 (7.8) | 4,057 (13.6) |
| Asian | 18,945 (2.3) | 1,039 (3.5) |
| Hispanic | 18,630 (2.3) | 314 (1.1) |
| Other | 24,539 (3.0) | 861 (2.9) |
| Men, n (%) | 269,787 (33.3) | 16,477 (55.4) |
| Region of residence, n (%) |  |  |
| Northeast | 151,932 (18.8) | 5,369 (18.0) |
| North central | 195,989 (24.2) | 7,034 (23.6) |
| South | 306,169 (37.8) | 12,020 (40.4) |
| West | 155,216 (19.2) | 5,330 (17.9) |
| Medicare-Medicaid eligible/low-income subsidy, n (%) | 152,656 (18.9) | 5,481 (18.4) |
| Diabetes, n (%) | 176,265 (21.8) | 10,943 (36.8) |
| Chronic kidney disease, n (%) | 85,255 (10.5) | 10,204 (34.3) |
| Atrial fibrillation, n (%) | 39,045 (4.8) | 2,546 (8.6) |
| Hypertension, n (%) | 467,839 (57.8) | 23,693 (79.6) |
| Medication use, n (%) |  |  |
| Diuretics | 205,660 (25.4) | 11,421 (38.4) |
| Statin | 274,107 (33.9) | 11,981 (40.3) |
| Low/moderate-intensity statin† | 269,480 (98.3) | 11,794 (98.4) |
| High-intensity statin† | 4,627 (1.7) | 187 (1.6) |
| COX-2-selective NSAIDs | 17,620 (2.2) | 871 (2.9) |
| Non-COX-2-selective NSAIDs | 107,717 (13.3) | 6,405 (21.5) |
| Antigout medications |  |  |
| Allopurinol | 7,389 (0.9) | 12,530 (42.1) |
| Colchicine | 1,626 (0.2) | 3,496 (11.8) |
| Probenecid | 212 (0.0) | 542 (1.8) |
| Febuxostat | 159 (0.0) | 563 (1.9) |
| COX: cyclooxygenase; NSAID: nonsteroidal anti-inflammatory drug; SD: standard deviation.  * Based on each patient’s index date.  † Percentages are calculated among those taking a statin. | | |

**Supplemental Table 12.** Incidence rates and hazard ratios for heart failure, myocardial infarction and stroke hospitalization and all-cause mortality among Medicare beneficiaries with and without gout included in the analysis stratified by race/ethnicity.

|  | **Non-Hispanic black** | | **Non-Hispanic white** | |
| --- | --- | --- | --- | --- |
|  | **without gout**  **(n=63,255)** | **with gout**  **(n=4,057)** | **without gout**  **(n=683,937)** | **with gout**  **(n=23, 482)** |
| **Heart failure hospitalization** |  |  |  |  |
| Events / person-years | 1,883 / 197,999 | 259 / 13,318 | 16,555 / 2,653,757 | 1,154 / 93,585 |
| Incidence rate (95% CI)* | 9.5 (9.1, 9.9) | 19.5 (17.1, 21.8) | 6.2 (6.1, 6.3) | 12.3 (11.6, 13.0) |
| Hazard ratio (95% CI) |  |  |  |  |
| Model 1 | 1 (reference) | 1.92 (1.69, 2.19) | 1 (reference) | 1.70 (1.60, 1.81) |
| Model 2 | 1 (reference) | 1.91 (1.68, 2.18) | 1 (reference) | 1.72 (1.62, 1.83) |
| Model 3 | 1 (reference) | 1.54 (1.34, 1.76) | 1 (reference) | 1.30 (1.22, 1.38) |
| **Myocardial infarction hospitalization** |  |  |  |  |
| Events / person-years | 1,943 / 198,144 | 181 / 13,461 | 24,051 / 2,629,820 | 1,227 / 93,129 |
| Incidence rate (95% CI)* | 9.8 (9.4, 10.2) | 13.5 (11.5, 15.4) | 9.2 (9.0, 9.3) | 13.2 (12.4, 13.9) |
| Hazard ratio (95% CI) |  |  |  |  |
| Model 1 | 1 (reference) | 1.25 (1.07, 1.46) | 1 (reference) | 1.20 (1.14, 1.28) |
| Model 2 | 1 (reference) | 1.26 (1.08, 1.47) | 1 (reference) | 1.22 (1.15, 1.29) |
| Model 3 | 1 (reference) | 1.12 (0.95, 1.30) | 1 (reference) | 1.07 (1.01, 1.13) |
| **Stroke hospitalization** |  |  |  |  |
| Events / person-years | 2,276 / 196,937 | 172 / 13,447 | 18,934 / 2,641,577 | 899 / 93,756 |
| Incidence rate (95% CI)* | 11.6 (11.1, 12.0) | 12.8 (10.9, 14.7) | 7.2 (7.1, 7.3) | 9.6 (9.0, 10.2) |
| Hazard ratio (95% CI) |  |  |  |  |
| Model 1 | 1 (reference) | 1.02 (0.87, 1.19) | 1 (reference) | 1.16 (1.09, 1.24) |
| Model 2 | 1 (reference) | 1.01 (0.87, 1.18) | 1 (reference) | 1.17 (1.09, 1.25) |
| Model 3 | 1 (reference) | 0.98 (0.83, 1.14) | 1 (reference) | 1.08 (1.00, 1.15) |
| **All-cause mortality** |  |  |  |  |
| Events / person-years | 11,515 / 201,745 | 954 / 13,806 | 126,755 / 2,684,663 | 5,715 / 95,738 |
| Incidence rate (95% CI)* | 57.1 (56.0, 58.1) | 69.1 (64.7, 73.5) | 47.2 (47.0, 47.5) | 59.7 (58.2, 61.2) |
| Hazard ratio (95% CI) |  |  |  |  |
| Model 1 | 1 (reference) | 1.04 (0.97, 1.11) | 1 (reference) | 1.03 (1.00, 1.05) |
| Model 2 | 1 (reference) | 1.03 (0.97, 1.11) | 1 (reference) | 1.05 (1.02, 1.08) |
| Model 3 | 1 (reference) | 0.96 (0.89, 1.02) | 1 (reference) | 0.96 (0.94, 0.99) |

**Supplemental Table 12.** *Continuation.*

|  | **Asian** | | **Hispanic** | |  |
| --- | --- | --- | --- | --- | --- |
|  | **without gout**  **(n=18,945)** | **with gout**  **(n=1,039)** | **without gout**  **(n=18,630)** | **with gout**  **(n=314)** | **p-value**† |
| **Heart failure hospitalization** |  |  |  |  |  |
| Events / person-years | 272 / 67,486 | 35 / 3,775 | 334 / 59,345 | 7 / 1,155 | - |
| Incidence rate (95% CI)* | 4.0 (3.6, 4.5) | 9.3 (6.2, 12.3) | 5.6 (5.0, 6.2) | 6.1 (1.6, 10.6) | - |
| Hazard ratio (95% CI) |  |  |  |  |  |
| Model 1 | 1 (reference) | 1.90 (1.33, 2.71) | 1 (reference) | 0.99 (0.47, 2.09) | 0.16 |
| Model 2 | 1 (reference) | 1.92 (1.34, 2.75) | 1 (reference) | 1.00 (0.47, 2.11) | 0.22 |
| Model 3 | 1 (reference) | 1.41 (0.97, 2.03) | 1 (reference) | 0.77 (0.36, 1.63) | 0.07 |
| **Myocardial infarction hospitalization** |  |  |  |  |  |
| Events / person-years | 510 / 66,993 | 42 / 3,742 | 475 / 59,078 | 7 / 1,158 | - |
| Incidence rate (95% CI)* | 7.6 (7.0, 8.3) | 11.2 (7.8, 14.6) | 8.0 (7.3, 8.8) | 6.1 (1.6, 10.5) | - |
| Hazard ratio (95% CI) |  |  |  |  |  |
| Model 1 | 1 (reference) | 1.16 (0.84, 1.60) | 1 (reference) | 0.65 (0.31, 1.37) | 0.41 |
| Model 2 | 1 (reference) | 1.16 (0.84, 1.59) | 1 (reference) | 0.65 (0.31, 1.37) | 0.39 |
| Model 3 | 1 (reference) | 0.92 (0.67, 1.28) | 1 (reference) | 0.52 (0.24, 1.10) | 0.20 |
| **Stroke hospitalization** |  |  |  |  |  |
| Events / person-years | 505 / 66,922 | 43 / 3,751 | 411 / 59,094 | 14 / 1,153 | - |
| Incidence rate (95% CI)* | 7.6 (6.9, 8.2) | 11.5 (8.0, 14.9) | 7.0 (6.3, 7.6) | 12.2 (5.8, 18.5) | - |
| Hazard ratio (95% CI) |  |  |  |  |  |
| Model 1 | 1 (reference) | 1.29 (0.94, 1.77) | 1 (reference) | 1.54 (0.90, 2.63) | 0.24 |
| Model 2 | 1 (reference) | 1.29 (0.94, 1.77) | 1 (reference) | 1.55 (0.91, 2.64) | 0.21 |
| Model 3 | 1 (reference) | 1.25 (0.91, 1.73) | 1 (reference) | 1.38 (0.81, 2.37) | 0.36 |
| **All-cause mortality** |  |  |  |  |  |
| Events / person-years | 2,164 / 68,013 | 187 / 3,855 | 2,560 / 60,014 | 48 / 1,177 | - |
| Incidence rate (95% CI)* | 31.8 (30.5, 33.2) | 48.5 (41.6, 55.5) | 42.7 (41.0, 44.3) | 40.8 (29.2, 52.3) | - |
| Hazard ratio (95% CI) |  |  |  |  |  |
| Model 1 | 1 (reference) | 1.12 (0.96, 1.30) | 1 (reference) | 0.81 (0.61, 1.07) | 0.26 |
| Model 2 | 1 (reference) | 1.12 (0.96, 1.30) | 1 (reference) | 0.80 (0.60, 1.07) | 0.23 |
| Model 3 | 1 (reference) | 1.01 (0.86, 1.18) | 1 (reference) | 0.76 (0.57, 1.02) | 0.40 |
| CI: confidence interval.  * Per 1,000 person-years.  † Comparing hazard ratios associated with gout across race/ethnic groups.  Model 1 includes adjustment for age and gender.  Model 2 includes adjustment for age, gender, region of residence, and Medicare-Medicaid eligible/low-income subsidy.  Model 3 includes adjustment for variables in Model 2 and diabetes, chronic kidney disease, atrial fibrillation, hypertension, and use of diuretics, statin (and statin intensity), and cyclooxygenase-2-selective and non-cyclooxygenase-2-selective nonsteroidal anti-inflammatory drugs. | | | | | |

**Supplemental Table 13.** Incidence rates and hazard ratios for heart failure, myocardial infarction and stroke hospitalization and all-cause mortality among Medicare beneficiaries with and without gout included in the analysis stratified by gender.

|  | **Men** | | **Women** | |  |
| --- | --- | --- | --- | --- | --- |
|  | **without gout**  **(n=269,787)** | **with gout**  **(n=16,477)** | **without gout**  **(n=539,519)** | **with gout**  **(n=13,276)** | **p-value**† |
| **Heart failure hospitalization** |  |  |  |  |  |
| Events / person-years | 5,728 / 1,005,237 | 633 / 64,804 | 13,679 / 2,059,615 | 844 / 50,324 | - |
| Incidence rate (95% CI)* | 5.7 (5.6, 5.9) | 9.8 (9.0, 10.5) | 6.6 (6.5, 6.8) | 16.8 (15.6, 17.9) | - |
| Hazard ratio (95% CI) |  |  |  |  |  |
| Model 1 | 1 (reference) | 1.48 (1.36, 1.61) | 1 (reference) | 1.96 (1.83, 2.10) | <0.001 |
| Model 2 | 1 (reference) | 1.52 (1.39, 1.65) | 1 (reference) | 1.95 (1.82, 2.09) | <0.001 |
| Model 3 | 1 (reference) | 1.19 (1.10, 1.30) | 1 (reference) | 1.45 (1.35, 1.56) | <0.001 |
| **Myocardial infarction hospitalization** |  |  |  |  |  |
| Events / person-years | 10,443 / 991,015 | 797 / 64,272 | 17,110 / 2,048,712 | 682 / 50,487 | - |
| Incidence rate (95% CI)* | 10.5 (10.3, 10.7) | 12.4 (11.5, 13.3) | 8.4 (8.2, 8.5) | 13.5 (12.5, 14.5) | - |
| Hazard ratio (95% CI) |  |  |  |  |  |
| Model 1 | 1 (reference) | 1.10 (1.02, 1.18) | 1 (reference) | 1.35 (1.25, 1.46) | <0.001 |
| Model 2 | 1 (reference) | 1.12 (1.04, 1.20) | 1 (reference) | 1.34 (1.24, 1.45) | <0.001 |
| Model 3 | 1 (reference) | 1.03 (0.95, 1.10) | 1 (reference) | 1.12 (1.04, 1.21) | 0.09 |
| **Stroke hospitalization** |  |  |  |  |  |
| Events / person-years | 7,001 / 999,382 | 576 / 64,698 | 15,656 / 2,050,966 | 576 / 50,659 | - |
| Incidence rate (95% CI)* | 7.0 (6.8, 7.2) | 8.9 (8.2, 9.6) | 7.6 (7.5, 7.8) | 11.4 (10.4, 12.3) | - |
| Hazard ratio (95% CI) |  |  |  |  |  |
| Model 1 | 1 (reference) | 1.13 (1.04, 1.23) | 1 (reference) | 1.16 (1.07, 1.27) | 0.63 |
| Model 2 | 1 (reference) | 1.15 (1.05, 1.25) | 1 (reference) | 1.16 (1.07, 1.26) | 0.89 |
| Model 3 | 1 (reference) | 1.09 (1.00, 1.19) | 1 (reference) | 1.05 (0.97, 1.15) | 0.55 |
| **All-cause mortality** |  |  |  |  |  |
| Events / person-years | 46,421 / 1,015,855 | 3,447 / 66,038 | 99,202 / 2,085,540 | 3,566 / 51,864 | - |
| Incidence rate (95% CI)* | 45.7 (45.3, 46.1) | 52.2 (50.5, 53.9) | 47.6 (47.3, 47.9) | 68.8 (66.5, 71.0) | - |
| Hazard ratio (95% CI) |  |  |  |  |  |
| Model 1 | 1 (reference) | 0.96 (0.93, 0.99) | 1 (reference) | 1.09 (1.06, 1.13) | <0.001 |
| Model 2 | 1 (reference) | 1.00 (0.97, 1.04) | 1 (reference) | 1.09 (1.06, 1.13) | <0.001 |
| Model 3 | 1 (reference) | 0.92 (0.89, 0.96) | 1 (reference) | 0.99 (0.96, 1.02) | 0.01 |
| CI: confidence interval.  * Per 1,000 person-years.  † Comparing hazard ratios associated with gout among men and women.  Model 1 includes adjustment for age and race/ethnicity.  Model 2 includes adjustment for age, race/ethnicity, region of residence, and Medicare-Medicaid eligible/low-income subsidy.  Model 3 includes adjustment for variables in Model 2 and diabetes, chronic kidney disease, atrial fibrillation, hypertension, and use of diuretics, statin (and statin intensity), and cyclooxygenase-2-selective and non-cyclooxygenase-2-selective nonsteroidal anti-inflammatory drugs. | | | | | |

**Supplemental Figure 1.** Flow-chart of REGARDS study participants included in the current analysis.

REGARDS: REasons for Geographic and Racial Differences in Stroke.

* Medicare fee-for-service inpatient and outpatient coverage was defined as having Medicare Parts A and B coverage without Part C coverage.

**Supplemental Figure 2.** Flow-chart of Medicare beneficiaries included in the current analysis**.**

US: United States.

* Medicare fee-for-service inpatient, outpatient and pharmacy coverage was defined as having Medicare Parts A, B and D coverage without Part C coverage.

† For each beneficiary, we selected a random index date between January 1, 2008 and June 30, 2015.

**Supplemental Figure 3.** Cumulative incidence of heart failure hospitalization with reduced and preserved left ventricular ejection fraction among REGARDS study participants with and without gout.

**
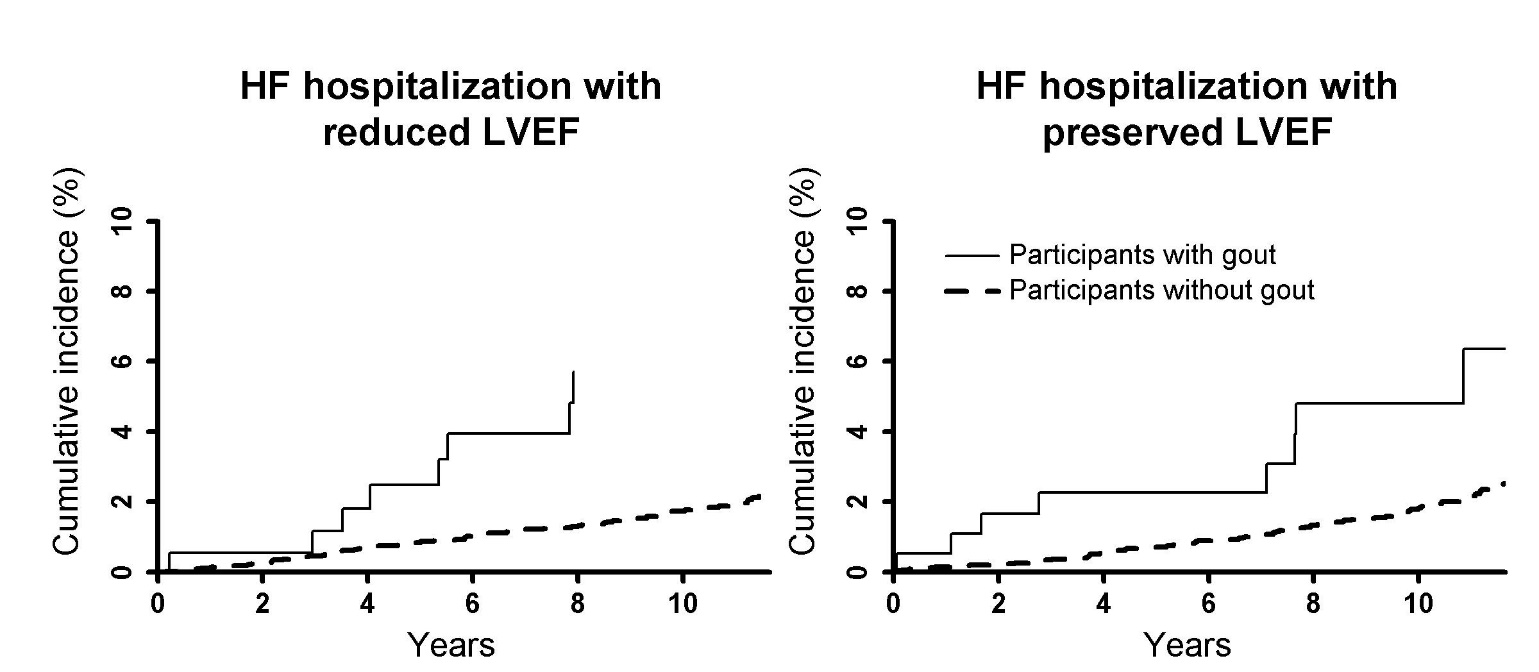
**

HF: heart failure; LVEF: left ventricular ejection fraction; REGARDS: REasons for Geographic And Racial Differences in Stroke.

Cumulative incidence curves are unadjusted.

**References**

1. Lara KM, Levitan EB, Gutierrez OM, Shikany JM, Safford MM, Judd SE, Rosenson RS. Dietary Patterns and Incident Heart Failure in U.S. Adults Without Known Coronary Disease. J Am Coll Cardiol. 2019;73(16):2036-2045.

2. Pullicino PM, McClure LA, Wadley VG, Ahmed A, Howard VJ, Howard G, Safford MM. Blood pressure and stroke in heart failure in the REasons for Geographic And Racial Differences in Stroke (REGARDS) study. Stroke. 2009;40(12):3706-3710.

3. Howard VJ, Cushman M, Pulley L, Gomez CR, Go RC, Prineas RJ, Graham A, Moy CS, Howard G. The REasons for Geographic And Racial Differences in Stroke Study: objectives and design. Neuroepidemiology. 2005;25(3):135-143.

4. Safford MM, Brown TM, Muntner PM, Durant RW, Glasser S, Halanych JH, Shikany JM, Prineas RJ, Samdarshi T, Bittner VA, Lewis CE, Gamboa C, Cushman M, Howard V, Howard G. Association of race and sex with risk of incident acute coronary heart disease events. JAMA. 2012;308(17):1768-1774.

5. Levey AS, Stevens LA, Schmid CH, Zhang YL, Castro AF, 3rd, Feldman HI, Kusek JW, Eggers P, Van Lente F, Greene T, Coresh J. A new equation to estimate glomerular filtration rate. Ann Intern Med. 2009;150(9):604-612.

6. Schneider KM, O'Donnell BE, Dean D. Prevalence of multiple chronic conditions in the United States' Medicare population. Health Qual Life Outcomes. 2009;7:82.

7. Kent ST, Safford MM, Zhao H, Levitan EB, Curtis JR, Kilpatrick RD, Kilgore ML, Muntner P. Optimal Use of Available Claims to Identify a Medicare Population Free of Coronary Heart Disease. Am J Epidemiol. 2015;182(9):808-819.

8. Kumamaru H, Judd SE, Curtis JR, Ramachandran R, Hardy NC, Rhodes JD, Safford MM, Kissela BM, Howard G, Jalbert JJ, Brott TG, Setoguchi S. Validity of claims-based stroke algorithms in contemporary Medicare data: REasons for Geographic And Racial Differences in Stroke (REGARDS) study linked with medicare claims. Circ Cardiovasc Qual Outcomes. 2014;7(4):611-619.

9. Singh JA. Veterans Affairs databases are accurate for gout-related health care utilization: a validation study. Arthritis Res Ther. 2013;15(6):R224.

10. Gandra SR, Lawrence LW, Parasuraman BM, Darin RM, Sherman JJ, Wall JL. Total and component health care costs in a non-Medicare HMO population of patients with and without type 2 diabetes and with and without macrovascular disease. J Manag Care Pharm. 2006;12(7):546-554.

11. Quan H, Sundararajan V, Halfon P, Fong A, Burnand B, Luthi JC, Saunders LD, Beck CA, Feasby TE, Ghali WA. Coding algorithms for defining comorbidities in ICD-9-CM and ICD-10 administrative data. Med Care. 2005;43(11):1130-1139.

12. Muntner P, Gutierrez OM, Zhao H, Fox CS, Wright NC, Curtis JR, McClellan W, Wang H, Kilgore M, Warnock DG, Bowling CB. Validation study of medicare claims to identify older US adults with CKD using the Reasons for Geographic and Racial Differences in Stroke (REGARDS) Study. Am J Kidney Dis. 2015;65(2):249-258.

13. Vlasschaert ME, Bejaimal SA, Hackam DG, Quinn R, Cuerden MS, Oliver MJ, Iansavichus A, Sultan N, Mills A, Garg AX. Validity of administrative database coding for kidney disease: a systematic review. Am J Kidney Dis. 2011;57(1):29-43.

14. Li Q, Glynn RJ, Dreyer NA, Liu J, Mogun H, Setoguchi S. Validity of claims-based definitions of left ventricular systolic dysfunction in Medicare patients. Pharmacoepidemiol Drug Saf. 2011;20(7):700-708.

15. Wilchesky M, Tamblyn RM, Huang A. Validation of diagnostic codes within medical services claims. J Clin Epidemiol. 2004;57(2):131-141.

16. Colantonio LD, Levitan EB, Yun H, Kilgore ML, Rhodes JD, Howard G, Safford MM, Muntner P. Use of Medicare Claims Data for the Identification of Myocardial Infarction: The Reasons for Geographic And Racial Differences in Stroke Study. Med Care. 2018;56(12):1051-1059.

17. Columbo JA, Kang R, Trooboff SW, Jahn KS, Martinez CJ, Moore KO, Austin AM, Morden NE, Brooks CG, Skinner JS, Goodney PP. Validating Publicly Available Crosswalks for Translating ICD-9 to ICD-10 Diagnosis Codes for Cardiovascular Outcomes Research. Circ Cardiovasc Qual Outcomes. 2018;11(10):e004782.

18. Traynor K. New gout treatment approved. Am J Health Syst Pharm. 2009;66(7):606.
